# FRETmatrix: a general methodology for the simulation and analysis of FRET in nucleic acids

**DOI:** 10.1093/nar/gks856

**Published:** 2012-09-12

**Authors:** Søren Preus, Kristine Kilså, Francois-Alexandre Miannay, Bo Albinsson, L. Marcus Wilhelmsson

**Affiliations:** ^1^Department of Chemistry, University of Copenhagen, Copenhagen, DK-2100, Denmark and ^2^Department of Chemical and Biological Engineering/Physical Chemistry, Chalmers University of Technology, S-41296 Gothenburg, Sweden

## Abstract

Förster resonance energy transfer (FRET) is a technique commonly used to unravel the structure and conformational changes of biomolecules being vital for all living organisms. Typically, FRET is performed using dyes attached externally to nucleic acids through a linker that complicates quantitative interpretation of experiments because of dye diffusion and reorientation. Here, we report a versatile, general methodology for the simulation and analysis of FRET in nucleic acids, and demonstrate its particular power for modelling FRET between probes possessing limited diffusional and rotational freedom, such as our recently developed nucleobase analogue FRET pairs (base–base FRET). These probes are positioned inside the DNA/RNA structures as a replacement for one of the natural bases, thus, providing unique control of their position and orientation and the advantage of reporting from inside sites of interest. In demonstration studies, not requiring molecular dynamics modelling, we obtain previously inaccessible insight into the orientation and nanosecond dynamics of the bases inside double-stranded DNA, and we reconstruct high resolution 3D structures of kinked DNA. The reported methodology is accompanied by a freely available software package, FRETmatrix, for the design and analysis of FRET in nucleic acid containing systems.

## INTRODUCTION

As cornerstones of the central dogma and fundamental players in gene regulation, nucleic acids and their structures, dynamics, conformational changes and interactions with other biomolecules is key to the understanding of living organisms. Traditionally, high-resolution structural insight into nucleic acids is accomplished using nuclear magnetic resonance (NMR) spectroscopy (1) or X-ray crystallography (2), often being complemented by lower-resolution techniques, such as Förster resonance energy transfer (FRET) (3,4). Of these, FRET possesses the prominent advantage of providing rapid measures of just a few nanomoles of sample in solution even in complex media or large molecular complexes. The method relies on the ability of a donor fluorophore to transfer its excitation energy to an acceptor chromophore through an oscillating transition dipole–dipole resonance mechanism. An inter-pair distance is obtained from the measured FRET efficiency provided that a reasonable value of the orientation factor, *κ*^2^, can be estimated based on previous knowledge of the system (equations explaining the relationship between transfer efficiency and distance as well as orientation can be found in the ‘Materials and Methods’ section) (5). Because of limitations in available dyes (6), by far the most FRET experiments use external fluorophores being tethered to the nucleic acid through a linker, thus, introducing dye diffusion and reorientation hampering the interpretation of quantitative experiments (7–9). Although recent advances have progressed the modelling of linker flexibility in quantitative FRET measurements (10–13), external labelling will always be accompanied by an inherent limitation in the information obtainable from the technique.

**Figure 1.**
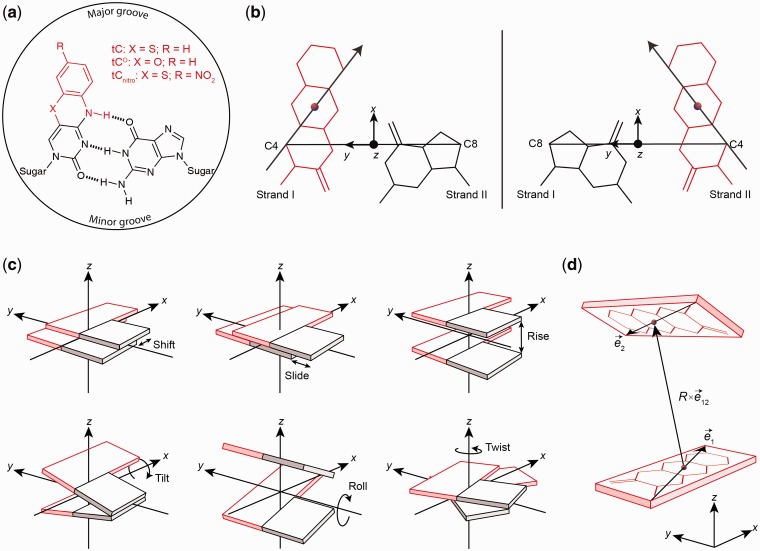
Using base probe building blocks for constructing 3D nucleic acid models and base–base FRET simulations. (**a**) Chemical structures of the tC base probes in their base pairing environment with guanine. tC and tC^O^ may act as FRET donor with tC_nitro_ as acceptor. (**b**) Definition of local tC–G base pair coordinate frame with tC in strand I (left) and strand II (right). In the base pair coordinate frame the *y*-axis is parallel to the line connecting the C4 atom of the tC base and the C8 atom of the complementary G base pointing from strand II to strand I. The *z*-axis points in the 5′→3′ direction of strand I, and the *x*-axis completes a right-handed set. The origin of the base pair frame is the midpoint of the C4–C8 line. (**c**) Definition of base pair step parameters used to construct 3D nucleic acid geometries. Shift, slide and rise are translational parameters, whereas tilt, roll and twist are rotational parameters. (**d**) Definition of 3D unit vectors used to simulate FRET in the constructed nucleic acid geometries.

We previously reported an all-nucleobase FRET pair system consisting of two, now commercially available, base analogues, tC^O^ and tC_nitro_ (Figure 1a) (14). These probes possess relatively stable photophysical properties in nucleic acid environments and are rigidly positioned inside the DNA/RNA structure mimicking the hydrogen bonding and base-stacking of natural cytosine (14–16) (manuscript in preparation regarding tC-family in RNA). The well-defined transition dipole position and orientation at the timescale of energy transfer is a considerable advantage of these FRET probes offering the potential to retrieve distance and orientational information from FRET measurements. Furthermore, the ability to position the reporters inside the very site of interest is an attractive, if not vital, feature in a majority of studies (17,18). However, the technical and theoretical challenges involved in the simulation and quantitative analysis of these probes have up till now posed serious limitations in the design and analysis of base–base FRET experiments. This calls for a diverse general methodology to simulate and analyse base–base FRET in any kind of nucleic acid structure.

Here, we report a general extendable methodology for the (i) simulation and (ii) quantitative analysis of FRET in nucleic acids. However, the method is particularly powerful for modelling constrained probes, including not only modified bases but also rigidly bound dyes (19–25). In the simulation part (i), an all-atom 3D nucleic acid model is constructed followed by a FRET simulation between static donor and acceptor dipole vectors. The generality of the implemented model building scheme makes the simulations completely independent of the geometrical shape of the nucleic acid allowing FRET in any structure to be modelled fast. To facilitate quantitative evaluation of FRET experiments in the analysis part (ii), we introduce user-defined directional probability distributions representing nanosecond rotational dynamics of the probes. The analysis routine performs a direct global fit of multiple time-resolved donor intensity decays from FRET experiments that increases the level of information obtainable from the measured data compared with analysing FRET efficiencies only. Depending on the objective of a given study, the analysis routine can easily be modified to search the data for local or global structural or dynamical features and provides a direct correlation between 3D nucleic acid structures and measured FRET signals.

The method is demonstrated experimentally by a combinatorial base–base FRET pairing approach, in which multiple probe positions are combined to gain quantitative information about the structure and the dynamics of the nucleic acid. In demonstration Study 1, the method is used to probe the local orientation and rotational fluctuations of the bases inside double-stranded DNA in solution. The second experimental study demonstrates how base–base FRET in combination with FRETmatrix can be used to reconstruct the 3D structure of local kinks, such as protein/ligand binding sites, bulges, junctions and DNA lesions. Here, we study two model structures allowing us to gauge our method against previous results and find excellent agreement. The information gained using the presented method is otherwise obtainable only hypothetically by means of much more complex and time-consuming NMR or single-molecule methods; thus, the method shows great promise for future innovative investigations. All of the reported methodology is accompanied by a MATLAB-based software package, FRETmatrix, equipped with a user-interface and freely available from http://www.chalmers.se/chem/EN/divisions/physical-chemistry/staff/marcus-wilhelmsson/fretmatrix.

## MATERIALS AND METHODS

### Building nucleic acid geometrical models

FRETmatrix implements a matrix-based base-centred calculation scheme [the Cambridge University Engineering Department Helix Computation Scheme (CEHS)] to build nucleic acid geometries (26). The scheme uses a standardized reference frame for the description of nucleic acid structures, in which the geometry of all base pairs and base pair steps in the structure are characterized by a set of rigid body parameters as described in detail previously (27–29). The reference frame definitions associate each base and each base pair with a local coordinate frame in which the Cartesian coordinates of all *n* atoms are predefined and stored in coordinate matrices:
(1)
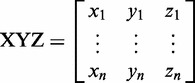

Building a 3D geometrical model is then accomplished by a set of matrix operations serving to position each local coordinate frame within the global coordinate frame of the structure. Such rotations are accomplished using the three rotation matrices:
(2)
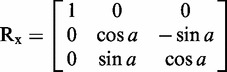

(3)
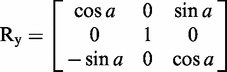

(4)
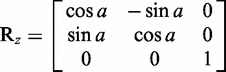

For example, if we wish to rotate a set of coordinates arranged in an **XYZ** matrix (equation 1) through an angle *a* about the *x*-axis, this is analogues to the matrix product:
(5)
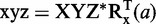

where R_x_^T^ is the transpose of the rotation matrix R_x_. Translating the transformed coordinates by a vector v_xyz_, arranged in an *n* × 3 matrix v, is then accomplished by
(6)
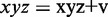

to yield the final set of 3D coordinates *xyz* defining the new position and orientation of the body. Using equations (1–6), one can construct and orient any geometrical body defined in three dimensions. In particular, building nucleic acid geometries is accomplished by rotating and translating the local base and/or base pair coordinate frames into the global nucleic acid coordinate frame. For example, going from one base pair to the next is accomplished by three consecutive rotations (tilt, roll and twist) and a three-coordinate translation (shift, slide and rise). See El Hassan and Calladine (26) and Lu *et al.* (27) for additional descriptions of the matrix-based equations used in CEHS.

#### Building geometries containing FRET probes

Based on the detailed information we have previously obtained regarding the structural and electronic properties of the tC bases (15,30,31), we can implement these base probes within the standardized nucleic acid reference frame and, thus, the CEHS model building scheme. We define the local tC base coordinate frame directly analogously to the pyrimidine base frame, and the local base pair coordinate frame of a synthetic tC-G base pair directly analogously to the corresponding C-G pair (Figure 1b and Supplementary Figure S1). The vectorial representations of the transition dipole moments of the probes are similarly defined within the local coordinate frames allowing the dipole positions and orientations to be unambiguously specified relative to the atomic coordinates of the base pair (Supplementary Tables S1 and S2).

Defining the local coordinate frame of a synthetic base allows us to simulate the exact position and orientation of probes positioned in any 3D nucleic acid structure by building up a geometrical model using the standard structural parameters to describe each dinucleotide step in the structure (Figure 1c). The result of this model building routine is a global coordinate matrix describing the position of all atoms and the positions and directions of the 3D transition dipole vectors of FRET pairs positioned in the structure.

#### Building two structural units separated by a kink

Building geometrical models consisting of multiple joined structural modules, such as two B-DNA helices separated by a kink, is achieved by FRETmatrix in a similar manner as constructing a base pair step namely using three Euler angles and a translation vector to describe the exact relative orientation and position of the two units. In FRETmatrix, the first base pair of unit 1 is defined as the global coordinate frame of the structure. The second unit, i.e. the second helix, is initially built within the coordinate frame of the first base pair of unit 2 and is then subsequently rotated and translated according to the specified kink parameters and is finally aligned with the last base pair of the previous unit using equations (1–6). Using this approach, an unlimited number of structural units can be joined.

### Simulating FRET in 3D nucleic acid structures

In short, the FRET efficiency between a FRET pair positioned in the geometrical model is given by
(7)
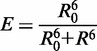

where the critical Förster distance (the distance at which *E* = 0.5) is calculated as
(8)
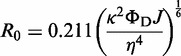

Here, Φ_D_ is the donor quantum yield in absence of acceptor, *J* is the spectral overlap integral between the donor emission and acceptor absorption spectrum and *η* is the refractive index, all being parameters exploited as previous knowledge. With the global coordinate matrix at hand, the two geometrical parameters, *κ* and *R*, are simulated using the extracted donor and acceptor dipole vectors: the orientation factor in the energy transfer process is calculated as a sum of vector products
(9)
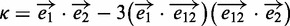

where 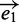 and 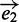 are the dipole unit vectors, and 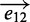 is the unit vector connecting the two dipole centres (Figure 1d), whereas the distance between two dipole centres, (*x*_1_, *y*_1_, *z*_1_) and (*x*_2_, *y*_2_, *z*_2_), is calculated as
(10)
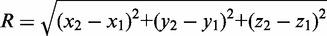

More detailed information on the calculation of FRET is provided in Supplementary Note S1.

### Modelling base dynamics

The simulations described above assume rigid nucleic acid geometries and static probe dipoles. The results of such first principle simulations are particularly useful for qualitative guiding purposes. In solution, nucleic acid dynamics occurring on the same timescales as the energy transfer process, i.e. from the sub picosecond range to the nanosecond regime, result in dipole orientational fluctuations during the excited state lifetimes of the probes. In ensemble measurements, the result is a distribution of donor–acceptor orientations, which should be implemented in the simulation to evaluate FRET experiments more quantitatively.

**Figure 2.**
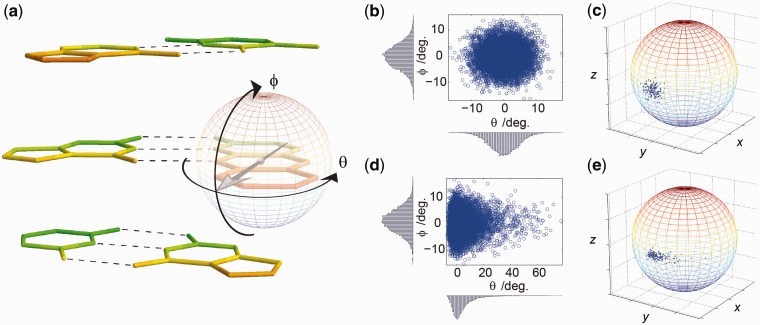
Simulating dipole vector directional distributions. (**a**) Definition of two spherical angles describing in-plane (θ) and out-of-plane rotation (ϕ) of a base probe in double-stranded DNA (only the nucleobases are shown). The two neighbouring base pairs are shown above and below the tC–G base pair. The strand in which the base probe is positioned has 5′ directed downwards and 3′ upwards. (**b–e**) Sampling dipole vectors from two marginal Boltzmann probability distributions. First, samples are drawn in spherical coordinates (b, d) and then subsequently transformed into Cartesian space where samples are located on the unit sphere representing dipole unit vectors (c, e). The form and width of the distribution in (b) and (d) is the same as in (c) and (e), respectively. In (b, c), a harmonic potential is used to describe in-plane and out-of-plane motion, corresponding to sampling a bivariate Gaussian distribution. In (d, e), a harmonic potential is used to describe out-of-plane motion, and a Lennard-Jones potential is used to describe the potential imposed by the in-plane hydrogen-bonding to the complementary base.

To model rotational dynamics of the probes, we extend the structure building scheme by including dipole vector directional distributions in the FRET simulation (Supplementary Note S2). First, it is recognized that the anisotropic microenvironment of the base probe when base pairing to its complementary base leads to the definition of two spherical coordinates representing two different modes of rotational movement of the base (Figure 2a). Here, the angle θ describes in-plane movement primarily being influenced by the potential energy imposed by the H-bonding to the complementary base. The angle ϕ, on the other hand, describes out-of-plane movement influenced by the base-stacking with neighbouring bases and internal bending modes of the structural framework of the probe. If the simulation is to be as physically accurate as possible, the directional probability distribution representing dipole reorientation should describe these fundamentally different characteristics along the θ- and ϕ-coordinates of the potential energy surface in which the base moves.

We model base fluctuations by assigning a 1D potential energy function to each of the two modes of dipole rotation. The corresponding Boltzmann distribution at room temperature then provides us with two 1D probability density functions, *P*_i_(θ) and *P*_o_(ϕ), describing the probability of finding the dipole vector along each of the two coordinates (Supplementary Figure S2). Here, the probabilities of θ and ϕ are independent, and the joint probability distribution over θ and ϕ is, therefore, given by
(11)
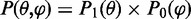



Although this equation accurately describes the probability distribution of the orientation of the base probe within a base stack, we have not been able to identify a closed analytical expression relating the energy transfer rate constant with expression (equation 11). The desired vector probability distribution is therefore implemented by sampling the joint probability distribution of *P*_i_ and *P*_o_ over each of the spherical coordinates (Figure 2b and d) and then subsequently transform the samples into Cartesian space (Figure 2c and e). To circumvent samples being piled up at the poles (Supplementary Figure S3), samples are initially drawn with a maximum likelihood of (θ*,*ϕ) = (0,0), i.e. in Cartesian space, the unit vector pointing in the (*x*,*y*,*z*) = (1,0,0) direction. The drawn samples are then subjected to a set of Euler rotations aligning the maximum likelihood of the distribution, with the direction of the transition moment vector of the probe positioned in its local coordinate frame. The distribution is put into place within the global nucleic acid structure in the same way as described earlier in the text for positioning a base pair. The resulting vector population, now representing the dipole orientational distribution traced during an energy transfer event, is used to calculate the corresponding energy transfer rate constant and simulate the donor decay. This stepwise construction of the dipole unit vector distribution ensures that the sampled vector distribution represents the potentials defined for the microenvironment of the probe, provided that the width of *P*_o_(ϕ) does not allow samples to be drawn near ϕ = ±90°. The form and width of the sampled dipole vector distribution directly reflect the base dynamics occurring on the timescale of the energy transfer, usually being in the range of 0.5–5 ns.

Assigning independent functional forms to the in-plane and out-of-plane rotational modes of the base probes makes the form and width of the simulated dipole vector distribution highly versatile (Supplementary Figure S4). For example, assigning harmonic potentials to θ and ϕ produces dipole vector distributions with appearances similar to the Kent or the Mises–Fisher directional distributions (Figure 2b and c), the analogues of the bivariate Gaussian distribution for directional data in three dimensions (10). To mimic the base pairing environment of a base probe, we model the in-plane movement using a Lennard-Jones potential (Figure 2d and e). This function first of all possesses the correct appearance expected for a hydrogen-bond and, secondly, is straight forward to model (Supplementary Figure S2). As shown below, the out-of-plane movement is almost negligible at the timescale of the energy transfer, making the choice of this functional form less important. For this reason, we use a harmonic potential to represent the out-of-plane movement of the base.

### Simulating donor intensity decays

As the entire time-resolved donor intensity decay contains several hundred more data points than the corresponding steady-state FRET efficiency, the amount of information obtainable from a FRET experiment is greatly increased by introducing the structural and dynamical parameters directly in the donor intensity decay fitting. We simulate the donor decays based on an exponential decay model that includes the Förster characteristics derived from the constructed nucleic acid geometry and associated dipole vector distributions. In the dynamic averaging regime the donor intensity decay in presence of FRET is
(12)
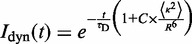

where 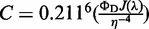
*, τ*_D_ is the donor lifetime in absence of acceptor, and *κ* and *R* are defined earlier in the text. Taking into account the potential presence of other fluorophores but the donor, the total decay is simulated as
(13)
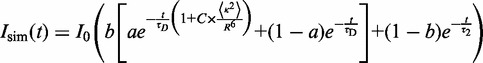

where *I*_0_ is a pre-exponential factor, (1−*a*) is the fraction of donors not coupled to an acceptor (Supplementary Figure S5), (1−*b*) is the fraction of emission from any other fluorophore but the donor, with a lifetime of *τ*_2_ (in our experiments being <5%). The simulated decay is convolved with the instrument response function of the time-correlated single photon counting measurement:
(14)
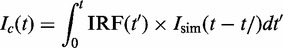

executed in FRETmatrix using the efficient fast Fourier transform-based method of overlap-add. The reduced *χ^2^* of the fit is then used to evaluate the goodness of the fit and is calculated as
(15)
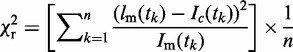

where *I*_m_*(t_k_*) is the number of measured counts at time *t*_k_, *I*_c_(*t*_k_) is the calculated number of counts at time *t*_k_, and *n* is the total number of datapoints (number of channels × number of samples) (32).

### Experimental details

All experiments were performed in phosphate buffer ([Na^+^] = 0.1 M) at *T* = 295 K. Donor concentrations were 0.5−2 µM, with 30% excess of the acceptor strand. Annealing was performed by heating up to 368 K followed by slow cooling to room temperature (RT) (12 h). Fluorescence lifetimes measurements were performed using time-correlated single photon counting. The excitation wavelength at 377 nm was delivered by a 10 MHz pulsed laser diode (PicoQuant). The fluorescence of the samples was spectrally filtered at 460 nm by a monochromator and detected by a thermoelectrically cooled microchannel plate photomultiplier tube Hamamatsu R3809U-66. The counts were sent to a multichannel analyser (Lifespec, Edinburgh Analytical Instruments) adjusted to 2048 channels, where a minimum of 10 000 counts were recorded in the top channel. The spectral resolution of the monochromator was fixed at 5 nm (emission slit width) and the time window was 100 ns. The instrument response function has a full width at half time maximum (FWHM) of 60 ps. For all FRET-pair positions, the lifetime measured of the donor-no-acceptor sample was used as donor reference.

#### Demonstration Study 1

One sequence motif was used to design three donor strands, each being complementary to four acceptor strands as described previously (Supplementary Table S3) (14). All probes were associated with identical directional vector distributions in the analysis. The in-plane movement was described by a Lennard-Jones potential defined by a dissociation energy of 0.1 eV, an experimentally determined average value of the three C–G hydrogen bonds (33,34). For the out-of-plane movement, we applied a harmonic potential. To obtain a low standard deviation in the calculation of 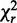, we sampled *N* = 1000 vectors when constructing the dipole distributions of each probe corresponding to 10^6^ dipole–dipole combinations of each FRET-pair resulting in a standard deviation of the calculated 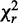 of 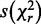 < 0.1 (Supplementary Figure S6). Increasing *N* for higher statistical significance is at the expense of increased computational time. All decays were analysed using equation (13) with experimentally determined input parameters (Supplementary Table S4).

#### Demonstration Study 2

Monomeric probes were purchased from Glen Research (Sterling, Virginia) and incorporated into DNA oligonucleotides and purified by reversed phase high-performance liquid chromatography (14) by ATDBio (Southampton, UK). DNA samples were designed to yield a large number of donor–acceptor combinations across the kinks with one global sequence motif for all samples (Supplementary Table S5). Donor decays from 18 FRET combinations of the 0A bulge and 16 combinations of the 3A bulge were combined and analysed using the six kink parameters as the only variables. To increase the number of FRET combinations, the design was made with the kink at two different positions in the sequence (only one kink per sample). In the analysis, the same set of kink parameters were used for both kink positions. Before beginning the FRET analysis, all decays were fitted to an exponential decay model providing decay parameters needed for the analysis (Supplementary Tables S6 and S7). All decays were then analysed using FRETmatrix and equation (13), using the experimentally determined input parameters (Supplementary Table S8). All donor–acceptor pairs were associated with the same set of simulation parameters as used in demonstration Study 1 (see earlier in the text) and, in addition θ-FWHM = 13°, ϕ-FWHM = 2°, ϕ_A_ = 25°, ϕ_D_ = 8°.

Two geometrical constraints were included in the DNA kink analysis algorithm (Supplementary Note S3):

- Kink parameter values resulting in a sterical clash between two or more atoms were automatically discarded. Here, the atomic van der Waals radius was set to 1.3 Å.
- A maximum distance of 11 Å between two neighbouring bases positioned in the same strand was allowed. This structural constraint is imposed physically by the covalent bonds connecting the two neighbouring bases.

For the 0A structure parameter, values optimized with and without geometrical constraints were identical. For the 3 A structure parameter, values optimized with and without geometrical constraints were close to being identical (Supplementary Table S9).

### Construction of *χ^2^* surfaces

The calculation of 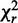 surfaces was built from 50−1600 point calculations depending on the parameter range. Interpolation was used to gain smooth surfaces. At each coordinate on the surface the parameters not being constrained in the analysis was optimized. Different algorithms were needed to optimize the parameters in the model. For parameters not associated with the random sampling process, a gradient-based algorithm with upper and lower boundary conditions was used. However, for ϕ-FWHM and θ-FWHM, the non-linear pattern search algorithm was chosen, as the random sampling step makes the object function non-differentiable. Optimizing kink parameters, including geometrical constraints, was best achieved using a two-step algorithm: an initial rough parameter search using the simulated annealing algorithm followed by a gradient-based optimization for determining the minimum more accurately. The confidence intervals of each fit parameter in the models were calculated based on the *F*-statistic using *P* = 0.05 (>95% chance that parameter value is consistent with data) (32). Running the 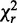 surfaces shown requires anything from a few minutes to several hours on a modern day laptop, depending on the size of the surface and the range of parameter values investigated.

### Software availability

The reported methodology is implemented in a MATLAB-based software package, which can be downloaded for free at http://www.chalmers.se/chem/EN/divisions/physical-chemistry/staff/marcus-wilhelmsson/fretmatrix. The program is designed for the preliminary design and subsequent quantitative analysis of experiments involving constrained FRET probes. A user guide is provided (Supplementary Note S3).

#### Implementing a new probe in FRETmatrix

To implement a new FRET probe in the methodology, the atomic coordinates of the base relative to the base reference frame must first be known and the direction of the transition dipole vector within the structural framework of the probe. A base pair building block is then constructed using a set of rigid body base pair parameters (stagger, stretch, shear, propeller, opening and buckle), the analogues of base pair step parameters (29). More detailed information, including a script constructing base pair building blocks from the atomic coordinates of a new base, is supplied with FRETmatrix (Supplementary Note S3). Examples of other dyes currently implemented in FRETmatrix are shown in Supplementary Figure S7.

## RESULTS

### Simulations of model geometries

#### Building model geometries

**Figure 3.**
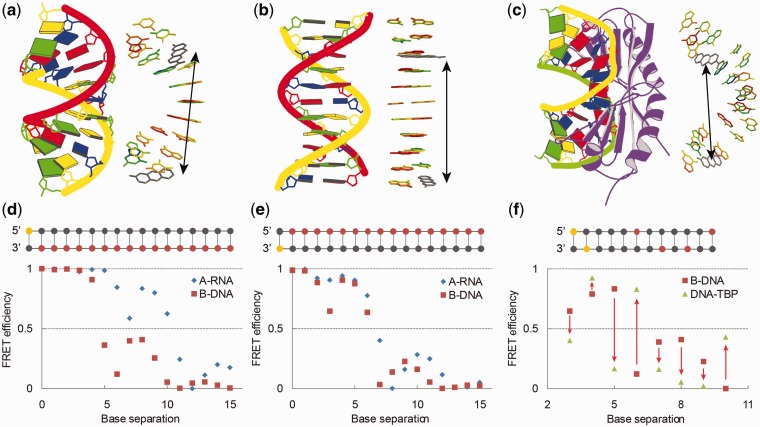
Demonstration of geometrical model building combined with FRET simulations in three model structures. (**a–c**) Representative examples of output geometries produced by FRETmatrix (right) along with the block representation of the corresponding structures produced by 3DNA (28) (left). Inserted FRET pairs are shown in grey and marked with arrows. The simulated structures are A-form RNA (a), B-form DNA (b), and PDB entry 1TGH: the complex between the TBP and DNA (c). (**d, e**) Simulated FRET efficiencies between tC^O^ and tC_nitro_ at selected positions within model structures (Supplementary Data S2). The positions of tC^O^ and tC_nitro_ in the strands are illustrated in top with the position of tC^O^ marked in yellow and the position of tC_nitro_ marked in red. Base separation denotes number of base pairs in between the FRET pair. In (**f**), red arrows denote change in FRET signal that would occur on binding of TBP to double-stranded DNA.

The versatility of the model building approach is demonstrated by three representative output structures from the FRETmatrix software (Figure 3a–c). FRETmatrix can build and gather regular A-form and B-form helices containing the FRET probes directly from an input sequence using base pair step parameters previously derived from experimentally determined structures (Figure 3a and b and Supplementary Table S10) (28,29). The program produces a Protein Data Bank (PDB) molecule file of the simulated structure for visual inspection purposes only. More complex nucleic acid structures, predefined in a PDB file, can be simulated through the use of a structural analysis routine that extracts all the structural parameters necessary to rebuild the geometrical arrangement of all bases in the structure (Supplementary Note S3) (28,35). The extracted structural parameters are then used as input for FRETmatrix that rebuilds a geometrical model of the structure inserting base probes at any desired positions. This rebuilding routine is demonstrated by PDB entry 1TGH, the complex formed between the TATA-binding protein (TBP) and its DNA target (Figure 3c and Supplementary Data S1) (36). In this form of simulation, the inserted FRET probe will possess the same geometrical position and orientation as the substituted base.

#### Simulating FRET in model geometries

The simulated FRET efficiencies of 16 base–base FRET combinations systematically positioned in standard A-form and B-form helices illustrate some of the advantages provided by the methodology presented here (Figure 3d and e). Firstly, the characteristic helical periodicity of A-form and B-form DNA are automatically seen as structural fingerprints without the need of imposing structure-dependent geometrical definitions and mathematical expressions relating the transition dipole vectors to the nucleic acid helical framework, such as in the still widely used model of Clegg *et al.* (37). Secondly, the method automatically takes into account any 5′/3′ effects, thus showing some insightful differences between the energy transfer efficiencies calculated for donor–acceptor pairs separated by the same distance but positioned 5′ (Figure 3d) versus 3′ ends (Figure 3e) on separate strands. These FRET differences are because of the difference in the relative orientation between donor and acceptor dipole vectors as can be concluded from the calculated FRET characteristics (Supplementary Data S2).

The high potential of base–base FRET to report on detailed structural changes of nucleic acids, e.g. on binding of a protein or other DNA ligands, is seen by comparing the simulated FRET signals at chosen donor–acceptor positions in regular B-DNA with the same set of donor–acceptor positions in the complex formed on binding of TBP (Figure 3f). These calculations predict the signal change that would occur when TBP binds to DNA, assuming the structural model is correct. The donor–acceptor positions chosen for this demonstrational simulation were selected from a screening of all possible FRET combinations, performed using an automated feature in FRETmatrix, with the criteria of yielding high signal changes on binding of substrate (Supplementary Note S3). Such simulations are particularly useful in studies requiring high throughput, e.g. for complementing higher resolution structural techniques when comparing different protein homologues bound to DNA. The high change in FRET signal at these positions is a combination of a change in donor–acceptor distance and, importantly, a change in the relative orientation between donor and acceptor (Supplementary Data S2).

### Demonstration Study 1

**Figure 4.**
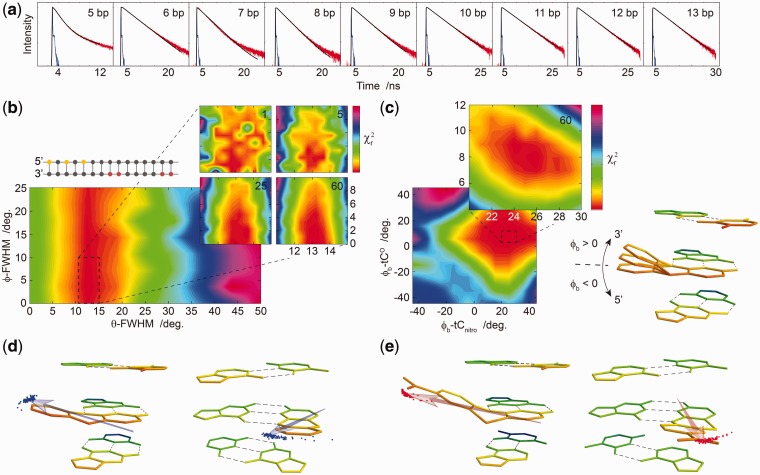
Using quantitative base–base FRET to obtain information about the orientation and nanosecond dynamics of the base probes in DNA. (**a**) Globally fit decays using the four optimized parameters. (**b**) Calculated 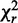 surface of the width of the directional distribution in the in-plane direction (θ-FWHM) and the out-of plane direction (ϕ-FWHM). Insert (left) illustrates the positions of donor and acceptor in the samples with tC^O^ marked in yellow and tC_nitro_ in red. In the experiment, only one donor–acceptor pair is positioned in each sample corresponding to nine different donor decays. Colour bar for large surface: 3.5:42. Insert (right) shows calculated 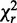 surface averaged from 1, 5, 25 and 60 simulations (colour bar: 3.5:4.3). (**c**) Calculated 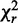 surface of the dipole bending angle of tC^O^ (ϕ_b_-tC^O^) and tC_nitro_ (ϕ_b_-tC_nitro_). Colour bar: 3.5:170. Insert (upper) shows averaged *χ^2^* surface after 60 simulations (colour bar: 3.5:5.5). Insert (right) illustrates the definition of ϕ_b_. The model shows a base probe in B-DNA where the 3′ end of the strand in which the probe is positioned points upward and 5′ is pointing downwards. Only the nucleobases are shown. (**d, e**) Illustration of the distribution made with the optimized parameters viewed from the side (left) and from the front (right) of tC^O^ (d) and tC_nitro_ (e).

To demonstrate experimentally how FRETmatrix and base–base FRET can be used to extract quantitative information on local nucleobase dynamics, we performed a global analysis of nine combinations of the tC^O^–tC_nitro_ pair positioned in B-DNA with distances varying from 5–13 base pairs (Figure 4b insert and Supplementary Table S3). As the overall helical structure of this model system is already well known, this allows us to analyse the data in terms of the geometry and directional fluctuations of the base probes in their base pairing environment with guanine. Besides demonstrating the power of the method, this study additionally provides information that can be exploited as previous knowledge in studies where other structural features of the nucleic acid are being probed as shown later in the text.

For this study, we defined four unknown parameters to be analysed while leaving all other variables constrained based on previous knowledge (Supplementary Tables S4 and S10). Two parameters describe the nanosecond dynamics of the probes, namely the FWHM of the directional distributions representing in-plane and out-of-plane base fluctuations. We additionally recognized the out-of-plane dipole bending angle, ϕ_b_, of tC^O^ and tC_nitro_ as two unknown variables of the system (Figure 4c insert). This geometrical parameter was introduced based on previous theoretical calculations suggesting that the tricyclic framework of the tC probes is highly flexible in terms of out-of-plane bending along the middle S-N axis of tC/tC_nitro_ or the O-N axis of tC^O^ (38). In there, we proposed a model in which the structure of the tC bases is energetically guided into a conformer characterized by bending into the major groove as a result of steric interactions with the 5′ neighbour. Confirming/disconfirming this model not only demonstrates the power of the method but also provides valuable insight into the properties of the tC probes in confined biological environments.

As the object function of our simulation, being the fit between measured and theoretical intensity decays, is dependent on random sampling, optimizing the parameters of the model is not achievable using a traditional gradient-based algorithm. Instead of performing an automated parameter optimization, we systematically investigate the entire parameter space relating all parameter values with the goodness of the global fit. The resulting 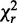 surfaces not only reveal the optimal parameter values but also provide a direct visualization of the uniqueness of the best fit and an estimation of the associated confidence intervals (Figure 4b and c). To overcome the inherent variations of the calculated 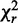 as a result of the implemented sampling procedure (Supplementary Figure S6), the simulation is performed several times averaging the calculated 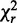 values in the final plot (Figure 4b and c inserts).

From this 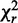 analysis routine, a single deep minimum is revealed on the 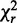 surface (Figure 4b and c) with optimal parameter values of θ-FWHM = 13.1° ± 0.3°, ϕ-FWHM < 2.5°, ϕ_b_-tC^O ^= 8.2 ± 1.7° and ϕ_b_*-*tC_nitro_ = 25° ± 2.5°. Visualizing the dipole vector distribution corresponding to these parameter values reveals a highly constrained orientational freedom of the base probes inside double-stranded DNA (Figure 4d and e). The negligible out-of-plane movement of the bases at the timescale of the energy transfer provides a direct insight into the influence of base-stacking on the dynamics of nucleobases. Strikingly, the out-of-plane bending angles correspond to bending towards the 3′ end of the strand in which the probes are positioned, thus confirming the previously suggested model in which the tricyclic framework of the tC bases is guided into the major groove (38). The fitted dipole bending values are close to the folding angles of the tC bases being <10° and 26° for tC^O^ and tC_nitro_, respectively, predicted using density functional theory (38).

### Demonstration Study 2

**Figure 5.**
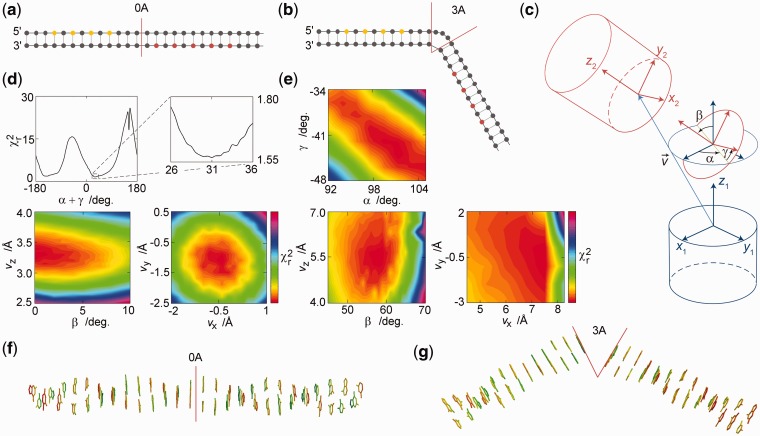
Using quantitative base–base FRET to reconstruct the 3D structure of nucleic acids. (**a**) Model system 1: a regular base pair step simulated as a local site in B-DNA. Donor positions are in yellow and acceptor positions in red. (**b**) Model system 2: a three adenine bulge. (**c**) Definition of kink parameters. The two helical coordinate systems are the base pair coordinate frames of the two base pairs neighbouring the kink. (**d**) 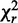 surfaces of the 0A system based on the global analysis of 18 donor decays (Supplementary Figure S9). Colour bars: 1.55:1.97 (left) and 1.55:1.78 (right). (**e**) Global analysis of the 3A bulge system based on the global analysis of 16 donor decays (Supplementary Figure S10). Figures show only the global minimum on the 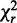 surface (Supplementary Figure S11). Colour bars: 1.85:2.12 (top), 1.85:2.39 (bottom left), 1.85:2.90 (bottom right). (**f**) Optimized 0A structure. (**g**) Optimized 3A structure.

Using the measured emission decay data from 16–18 different positions of the tC^O^–tC_nitro_ FRET-pair, we demonstrate how base–base FRET together with FRETmatrix can be used experimentally to reconstruct 3D nucleic acid structures. The following two model systems are studied: a regular base pair step (0A bulge, Figure 5a) and a three adenine bulge (3A bulge, Figure 5b). A translation vector, 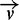, and three Euler angles with a *ZXZ* convention describe the relative position and orientation of the two structural units separated by the kink (Figure 5c), whereas the two helices themselves are modelled using base pair step parameters for regular B-DNA (Supplementary Table S10). Here, the 0A bulge constitutes the only model system that allows us to compare all six fitted kink parameters with known values, whereas mainly the DNA bending angle, β, of the 3A bulge can be compared with results from previous studies (12,39,40). In the analysis, the base probe bending angles and dipole distributions obtained from demonstration Study 1 were exploited as previous knowledge, allowing the DNA geometries to be probed with greater accuracy.

For both model structures, the results are in good agreement with expected geometries (Figure 5d–g). The optimized 0A kink parameter values are *v*_x_ = −0.55 ± 0.3 Å, *v*_y_ = −1.1 ± 0.3 Å, *v*_z_ = 3.3 ± 0.1 Å, α + γ = 31 ± 1°, β < 2° (Figure 5 d and f). All six 0A parameter values are thus in close agreement with the values for a regular base pair step with an accuracy of <1 Å for the translational shift and a few degrees for the angles of rotation. Notice that for β = 0, α and γ are directly correlated and display a sinusoidal influence on 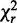 with a period of 180° (Figure 5d), as twisting the two helical units by an angle α + γ = 180° from an initial to a second state results in the dipole vectors being oppositely oriented but practically parallel as in the initial state.

The optimized 3 A kink parameter values are *v*_x_ = 7.4 ± 0.1 Å, *v*_y_ = −0.55 ± 0.6 Å, *v*_z_ = 5.5 ± 0.2 Å, α = 101 ± 2°, β = 57 ± 1°, γ = −43 ± 2° (Figure 5e and g). The 3A DNA bending angle of β = 57° ± 1° is in good agreement with previous studies using ensemble FRET (50°−70°) (39), transient electric birefringence (58° ± 4°) (40), and single-molecule FRET (56° ± 4°) (12). The all-atom 3A structure shows good overlap with the structure of another 3A bulge geometry obtained using single-molecule FRET with multiparameter fluorescence detection (Supplementary Figure S8) (12). The global fit between measured and theoretical intensity decays of all D-A separations is good for the 0A and 3A model systems (Supplementary Figures S9 and S10, respectively). It is noted that the full *χ^2^* surface oscillates with a period of α + γ = 180° (Supplementary Figure S11) as observed for the 0A bulge model system. The true global minimum was identified based on the assumption that DNA bending follows the right handed helical twist of the two helices (α + γ ≈ 55°).

## DISCUSSION

Compared with external labelling, base–base FRET offers a unique possibility to position the reporters inside the site of interest probing the local orientation and dynamics at specific base positions within nucleic acids. Global nucleic acid dynamics occurring at timescales exceeding the probe lifetimes are in principle also obtainable using the methodological framework presented here, although this was not demonstrated herein. The combined signal from several donor–acceptor positions can provide high-resolution distance and orientational information of nucleic acid structures without complications associated with fluorophore linker flexibility or DNA-dye interactions.

Various approaches have previously been developed to model probe dynamics during the energy transfer process (10–12,19). Importantly, our method, which uses user-defined directional distributions, provides a one-step analysis without the need to include force field molecular dynamics simulations in the analysis. The highly versatile form of the vector distribution is a particular advantage when modelling the orientationally constrained base probes in various nucleic acid environments. Using actual energy potentials to describe the nucleic acid dynamics paves the way for novel experimental studies of the fundamental physical properties of DNA and RNA structures.

It is recognized that there are two modes of dipole dynamics, reorientation and diffusion, being reflected in the energy transfer efficiency through the values of *κ*^2^ and *R*, respectively (12). Although dipole diffusion is pronounced when measuring FRET between external fluorophores, the tC base probes are rigidly positioned at relatively close distances inside the DNA structures. For this reason, we only modelled the orientational fluctuations of the probes. However, the method is expandable to include dipole diffusion when studying more dynamic structures.

In the demonstration studies, we used dynamic averaging of *κ*^2^, which assumes that the rotational correlation time of the probes is much faster than the energy transfer (5). This assumption was shown to be valid for external fluorophores (12) and is supported here by an internal correlation time of the base probes of *τ*_int_ = 350 ps estimated from the time-resolved fluorescence anisotropy decay of tC^O^ in high-viscosity solution (Supplementary Figure S12). In addition, the donor decays used in our studies are all well-fit using a single lifetime to represent FRET, which is a strong indication of dynamic averaging (Supplementary Table S6).

## SUMMARY AND OUTLOOK

We have developed a general methodological platform for simulating FRET in nucleic acids and demonstrated its particular power in modelling probes possessing limited degree of diffusional and rotational freedom. The method is based on the ability to rapidly construct any 3D nucleic acid geometry and simulate FRET between probes positioned anywhere within the structure. Directional vector distributions are implemented to model rotational dynamics of the probes, which, in combination with direct global intensity decay fitting of multiple donor and acceptor pairs, may provide quantitative information about structural and dynamical properties of nucleic acids. The method was used in combination with base–base FRET to obtain insight into base dynamics occurring on the timescale of energy transfer and to probe the exact 3D structure of kinked DNA in solution. Importantly, the method is versatile and expandable.

As a result of the rapidly progressing field of fluorescent nucleobase analogues (16–18) and other rigidly attached probes (19–25), including the popular Cy3–Cy5 pair shown to be partly constrained when tethered to the ends of nucleic acids (7,9,19,41,42), we anticipate that many fluorescent markers will be modelled in the future using the methodology presented here. Given the versatility of base–base FRET combined with the ready-to-use methodological platform reported here, we believe that new possibilities for experimental studies of nucleic acid structure and dynamics have opened up.

## SUPPLEMENTARY DATA

Supplementary Data are available at NAR Online: Supplementary Tables 1–10, Supplementary Figures 1–12, Supplementary Notes 1–3, Supplementary Data sets 1 and 2 and Supplementary References [1–13].

## FUNDING

Swedish Research Council (VR); Stiftelsen Olle Engkvist Byggmästare; Danish Council for Independent Research | Natural Sciences (FNU). Funding for open access charge: The Swedish Research Council (VR).

*Conflict of interest statement*. None declared.

## Supplementary Material

Supplementary Data
