# Supplementary material for: FRETmatrix: a general methodology for the simulation and analysis of FRET in nucleic acids
Source: Nucleic Acids Res. 2012 Sep 12;41(1):e18. doi: 10.1093/nar/gks856 (PMC3592456; doi:10.1093/nar/gks856)
Supplement: Supplementary Data [file supp_41_1_e18__index.html]

FRETmatrix: a general methodology for the simulation and analysis of FRET in nucleic acids — FRETmatrix: a general methodology for the simulation and analysis of FRET in nucleic acids — Supplementary Data 

# FRETmatrix: a general methodology for the simulation and analysis of FRET in nucleic acids

## Supplementary Data

files

**Files in this Data Supplement:**

- Supplementary Data - docx file
