# Supplementary material for: FRETmatrix: a general methodology for the simulation and analysis of FRET in nucleic acids
Source: Nucleic Acids Res. 2012 Sep 12;41(1):e18. doi: 10.1093/nar/gks856 (PMC3592456; doi:10.1093/nar/gks856)
Supplement: Supplementary Data [file supp_gks856_nar-01714-met-f-2012-File007.docx]

# FRETmatrix: A general methodology for the simulation and analysis of FRET in nucleic acids

**Søren Preus, Kristine Kilså, Francois-Alexandre Miannay, Bo Albinsson, and L. Marcus Wilhelmsson**

| **Supplementary Figure 1** | The position and direction of the transition dipole moments of tC, tC^O^ and tC_nitro_ in their base reference frames. |
| --- | --- |
| **Supplementary Figure 2** | Modelled potentials and corresponding Boltzmann distributions. |
| **Supplementary Figure 3** | Illustration of the pole pile up effect. |
| **Supplementary Figure 4** | Sampled vector distributions characterized by different widths. |
| **Supplementary Figure 5** | Effect of *a* on the simulated decay. |
| **Supplementary Figure 6** | Standard deviation of the reduced global chi-square as a function of the number of dipoles drawn in the simulation. |
| **Supplementary Figure 7** | Examples of other dyes but the tC bases implemented in FRETmatrix |
| **Supplementary Figure 8** | Optimized 3A structure overlay. |
| **Supplementary Figure 9** | Global fits of demonstration study 2 (0A bulge). |
| **Supplementary Figure 10** | Global fits of demonstration study 2 (3A bulge). |
| **Supplementary Figure 11** | Full α,γ chi-square surface of 3A bulge model system. |
| **Supplementary Figure 12** | Time-resolved fluorescence anisotropy of tC^O^ in double-stranded DNA. |
| **Supplementary Table 1** | Atomic coordinates and transition dipole coordinates of tC, tC^O^ and tC_nitro_ in the nucleobase coordinate frames. |
| **Supplementary Table 2** | Atomic coordinates of tC, tC^O^ and tC_nitro_ in the base-pair coordinate frame. |
| **Supplementary Table 3** | DNA sequences in demonstration study 1.Base-pair step parameters of regular A-form and B-form helices used by FRETmatrix. |
| **Supplementary Table 4** | Parameter values used in demonstration study 1. |
| **Supplementary Table 5** | DNA sequences in demonstration study 2. |
| **Supplementary Table 6** | Double stranded donor reference decays fit of demonstration study 2. |
| **Supplementary Table 7** | Donor decays in demonstration study 2 fit in presence of FRET. |
| **Supplementary Table 8** | Parameter values used in demonstration study 2. |
| **Supplementary Table 9** | Fitted kink parameter values of the 3A bulge. |
| **Supplementary Table 10** | Base-pair step parameters of regular A-form and B-form helices. |
| **Supplementary Note 1** | Simulating FRET using three dimensional dipole vector representations. |
| **Supplementary Note 2** | Flow chart for simulating dipole dynamics. |
| **Supplementary Note 3** | FRETmatrix v1.0 user guide. |
| **Supplementary Data 1** | Base-pair step parameter matrix of model DNA-TBP complex. |
| **Supplementary Data 2** | Simulated FRET characteristics of model systems. |

# SUPPLEMENTARY FIGURES


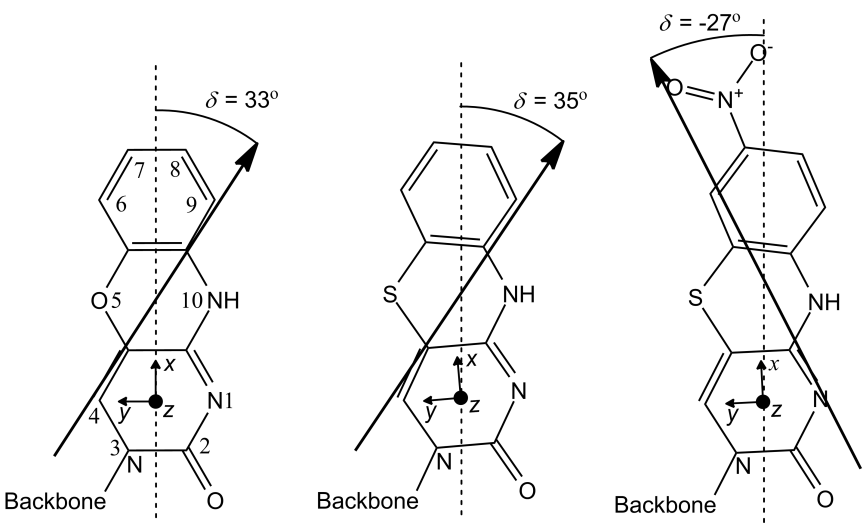


Supplementary Figure 1. The position and direction of the transition dipole moments of tC, tC^O^ and tC_nitro_ and their base reference frames being centred in the bottom ring system for the base positioned in strand I. The corresponding atomic coordinates and transition dipole coordinates are shown in Supplementary Table 1. The *y­-*axis of this frame is pointing towards the C4 atom, the *x*-axis is pointing in the direction of the outer ring and the *z*-axis is pointing perpendicularly to the base plane. For the base in strand II the coordinate frame is rotated 180° about the positive *x*-axis. Since the sulphur atom perturbs the symmetry of the tricyclic frameworks of tC and tC_nitro_, a small angular correction was made for the direction of the molecular long axis. The angle between the molecular long axis (dashed line) and the base reference frame *x*-axes are 0^0^, 5^0^, and 3^0^ for tC^O^, tC, and tC_nitro_, respectively. The dipole centers were set to be in the center of the mid ring of the tricyclic framework. Values of *δ* have been estimated experimentally for tC ([1](#_ENREF_1)), tC^O^ ([2](#_ENREF_2)), and tC_nitro_ ([3](#_ENREF_3)).


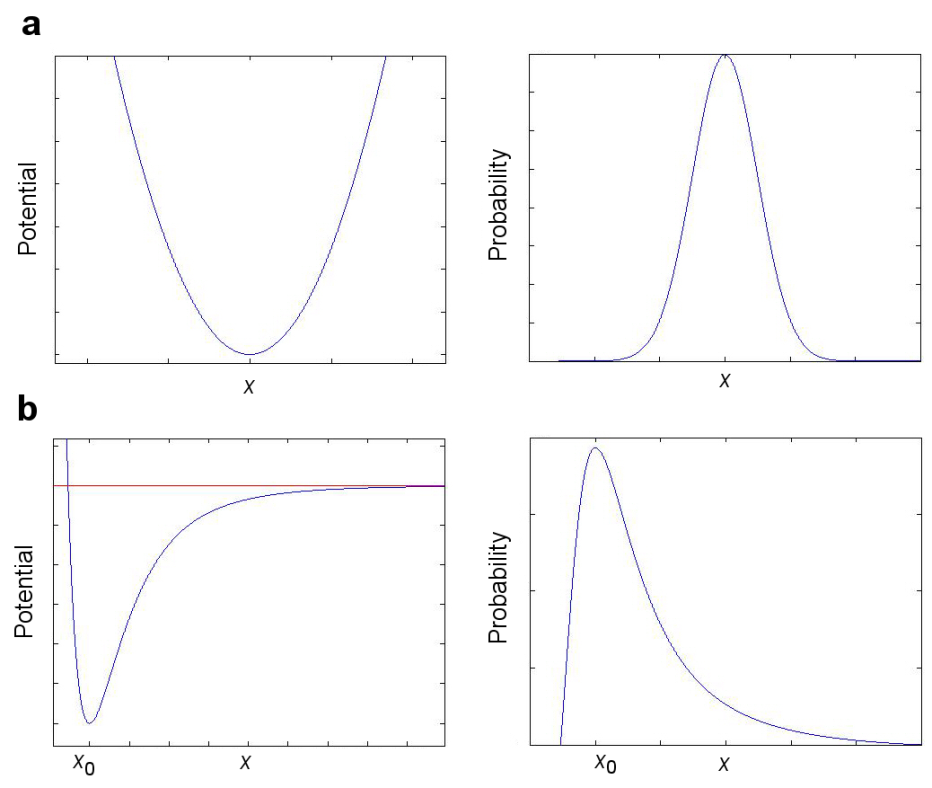


**Supplementary Figure 2.** Modeled potentials (left) and corresponding Boltzmann distributions (right). (a) Harmonic potential. (b) The Lennard Jones potential we use for the in-plane movement is given by

$$\boldsymbol{V}\left( \boldsymbol{x} \right)\boldsymbol{=D}\left[ \left( \frac{\boldsymbol{x}_{\boldsymbol{min}}}{\boldsymbol{x}} \right)^{\boldsymbol{12}}\boldsymbol{-2}\left( \frac{\boldsymbol{x}_{\boldsymbol{min}}}{\boldsymbol{x}} \right)^{\boldsymbol{6}} \right]$$

with a dissociation energy of *D* = 0.03 eV and a temperature of *T* 296 K. *V* has a minimum at *V*(*x*_min_). The Boltzmann probability distribution of a species in the energy potential *V* is then

$$P\left( x \right)=Ae^{\frac{-V(x)}{k_{B}T}}$$

where *A* is a normalization factor *k*_B_ is the Boltzmann constant and *T* is the temperature. Only points below the defined red threshold line are used when constructing the probability distribution.


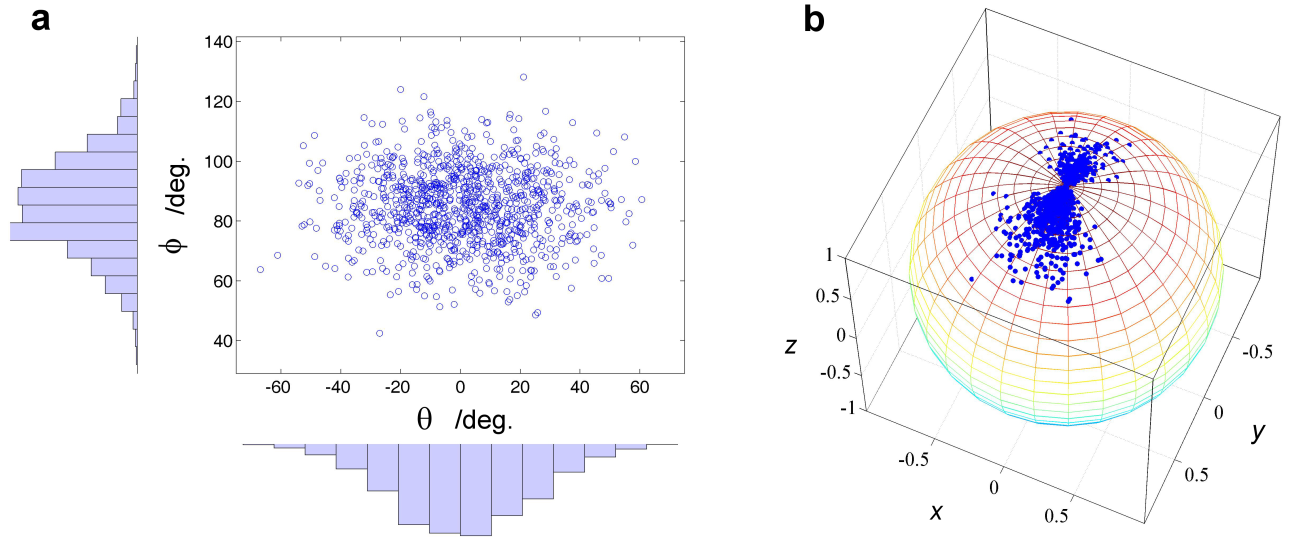


Supplementary Figure 3. When transforming sampled spherical coordinates into Cartesian coordinates, samples drawn near the poles of the unit sphere (i.e. near φ = ±90°) will pile up in Cartesian space compared to points sampled near the equator. (A) Scatter and histograms of samples drawn from a bivariate Gaussian distribution in spherical coordinates centered at θ = 0^0^ and φ = 85^0^. (B) Cartesian coordinates of the same samples as in (A) showing the pole pile up effect. The piling up of samples near the poles arise because of the smaller distance covered by a walk θ = 360^o^ around the sphere when walking near the pole (φ = 90^o^) compared to near the equator (φ = 0^o^).


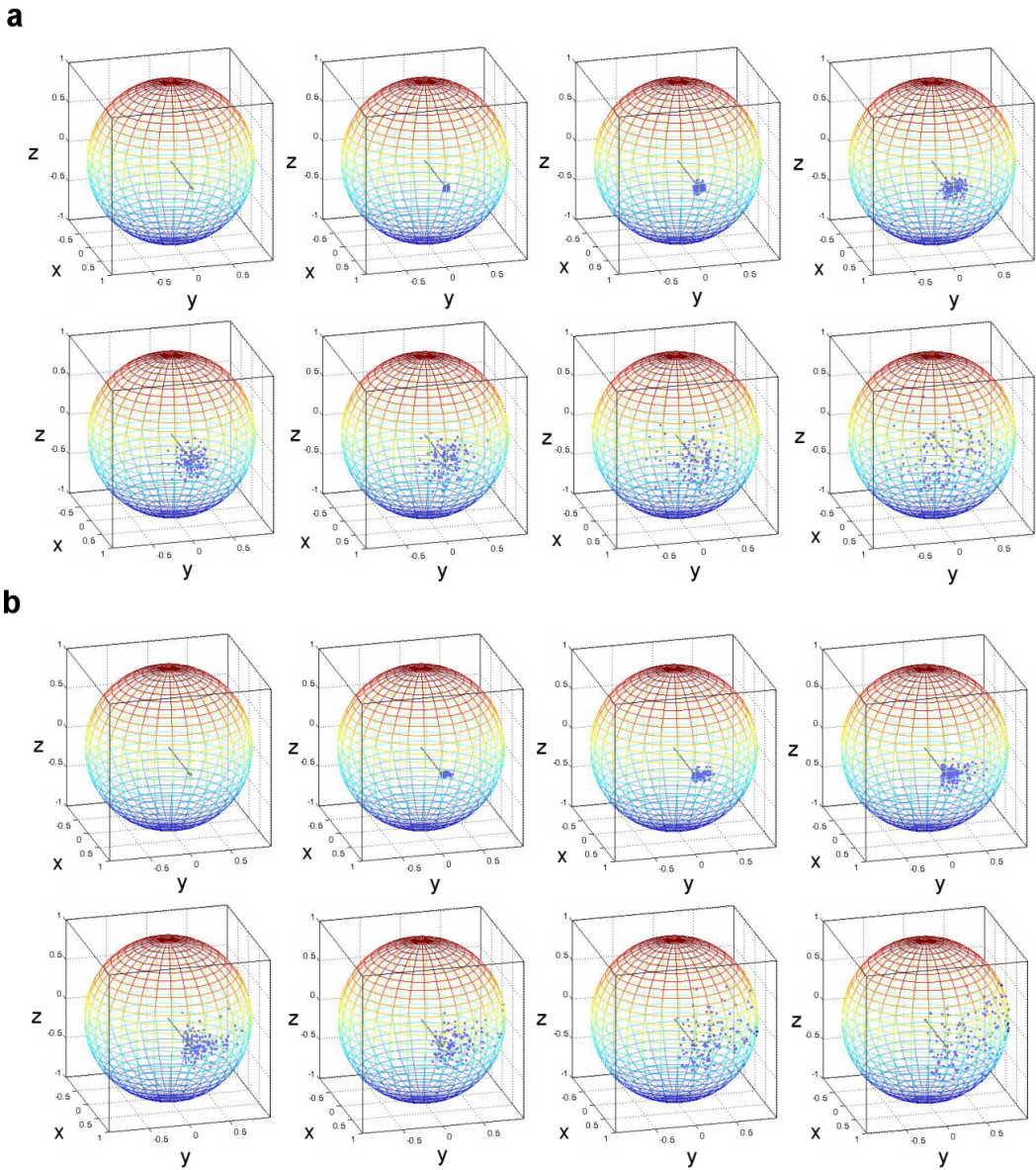


Supplementary Figure 4. Sampled vector distributions in Cartesian space characterized by different distribution widths. (a) Using a harmonic potential to describe both the φ and the θ coordinate which corresponds to sampling a bivariate Gaussian distribution. The same FWHM is used in both directions. Top row from left to right: FWHM = 0.1^0^, 2^0^, 5^0^, 10^0^. Bottom from left to right: FWHM = 15^0^, 20^0^, 30^0^, 40^0^. (b) Using a harmonic potential for the φ direction and a Lennard Jones potential for the θ direction. The FWHM for the two coordinates in each subfigure is the same as in (a).


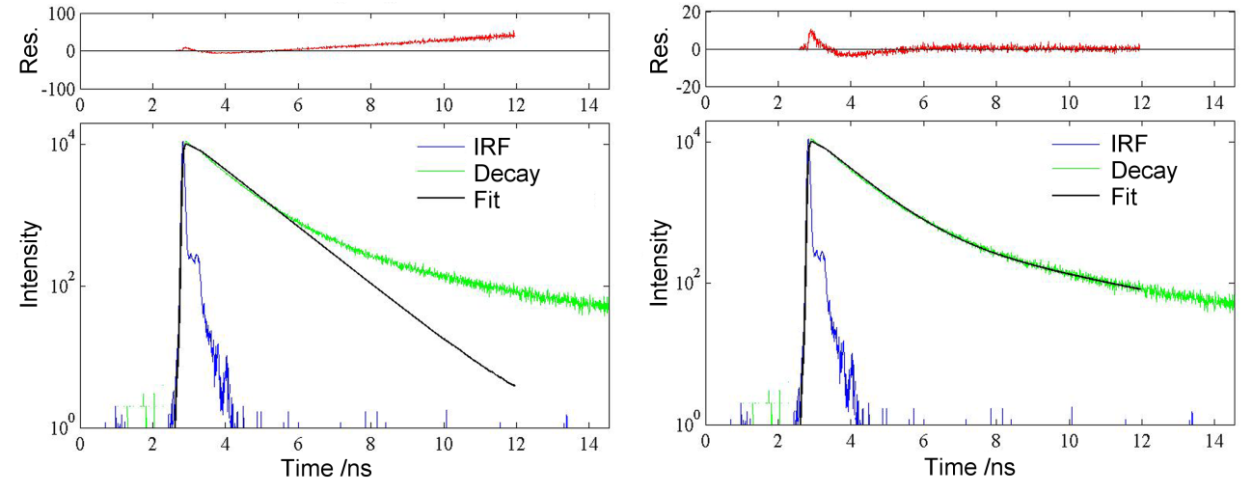


Supplementary Figure 5. Effect of *a* on the simulated decay. Left: *a* = 0.0000. Right: *a* = 0.0063 corresponding to 0.63% of donor fluorophores not coupled to an acceptor. Blue is the instrument response function (IRF), green is the measured decay, and black is the simulated decay convolved with the IRF. The example is a sample with a 5 base-pair separation between donor and acceptor. In both cases *b* = 0.


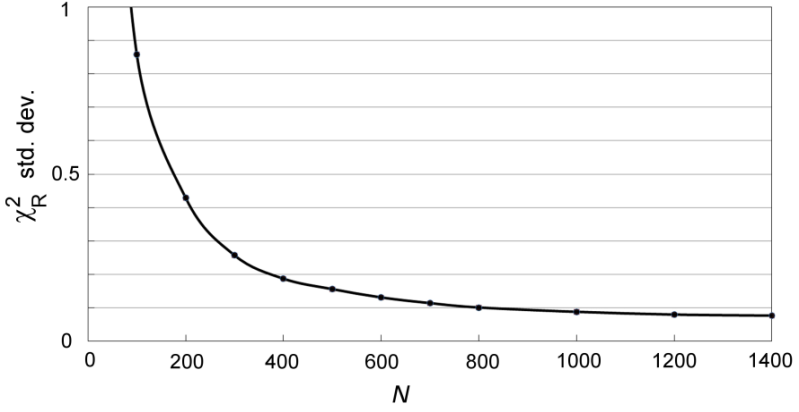


Supplementary Figure 6. Standard deviation of the reduced global chi-square as a function of the number of dipoles, *N,* drawn in the simulation. Since the object function is based on random sampling of the directional dipole probability distribution the accuracy of the simulation depends on the number of dipoles, *N*, drawn. The calculated $\boldsymbol{\chi}_{\mathbf{R}}^{\boldsymbol{2}}$ values are based on >100 repeated simulations of the data from demonstration study 1 when sampling from a distribution with *θ*-FWHM = 12^0^ and *φ*-FWHM = 4^0^.

Supplementary Figure 7. Examples of other dyes but the tC bases implemented in FRETmatrix (in the figure dyes are positioned in strand I). A) Fluorescein/rhodamine-based dyes tethered to thymine by a C6 linker. In FRETmatrix the dyes are positioned in their mean positions relative to the B-DNA helix as determined for the structurally similar Alexa 488 fluorophore by Sindbert *et al.* using MD simulations.([4](#_ENREF_4)) B) A Zn/2H porphyrin dye rigidly tethered to thymine through an acetylenic linker. The origin of the coordinate frame is the mid-point of the line connecting the C8 of adenine and C6 for thymine. The *y*-axis is parallel to the C8-C6 line pointing from strand II to strand I. The *x*-axis points into the major groove while the *z*-axis completes a right-handed set. The dipole centers are marked as black dots and are (x,y,z) = (0.86,12.69,5.06) and (x,y,z) = (11.94,8.37,0.0), respectively (in Å).


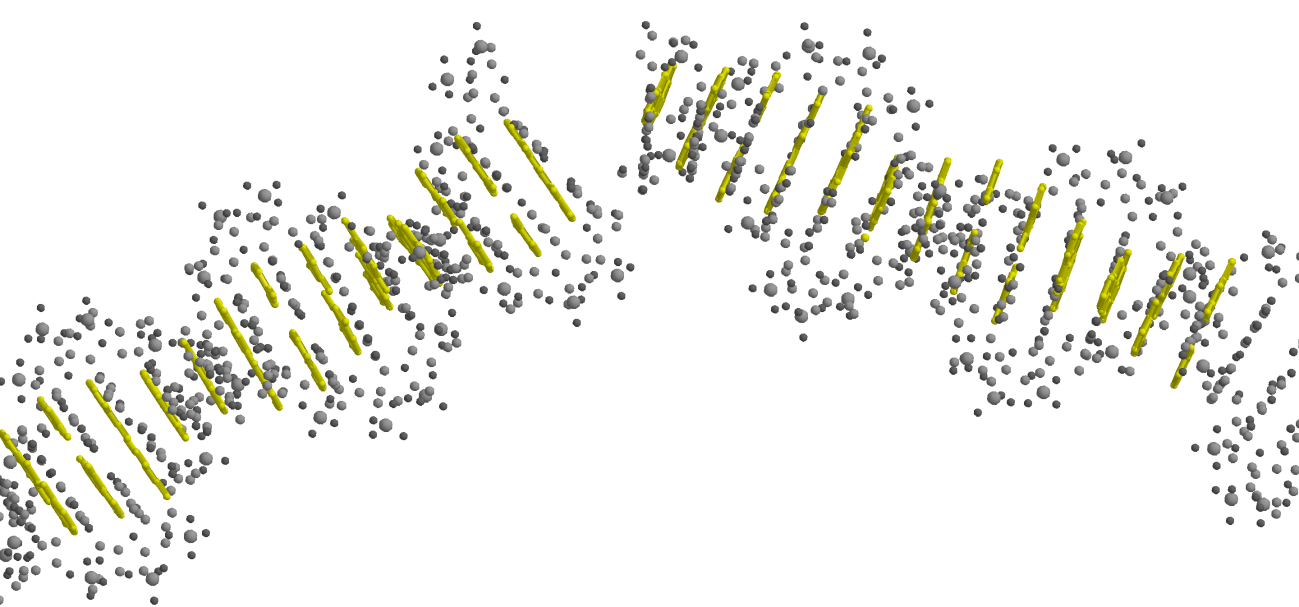


Supplementary Figure 8. Optimized 3A bulge structure (yellow) overlayed on the atomic framework of a 3A bulge structure obtained by Wozniak *et al.* using Multiparameter Fluorescence Detection Single-Molecule FRET (grey) ([5](#_ENREF_5)).

| 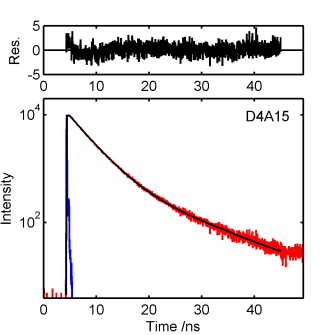 | 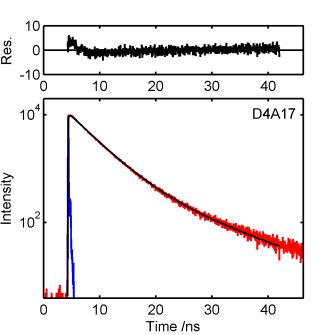 | 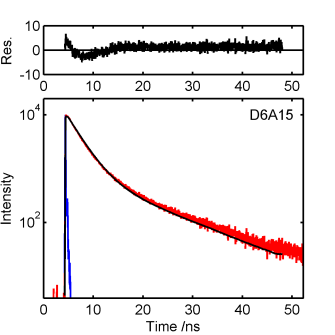 | 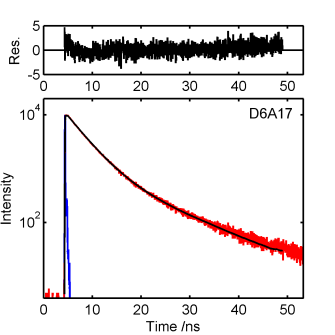 |
| --- | --- | --- | --- |
| 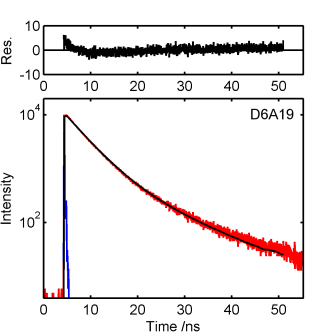 | 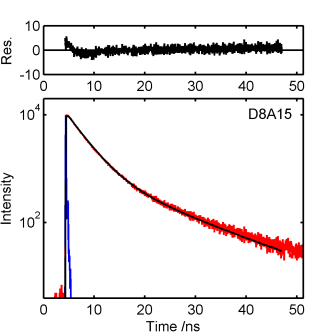 | 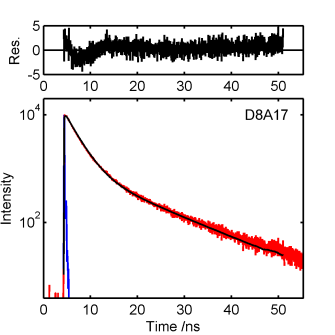 | 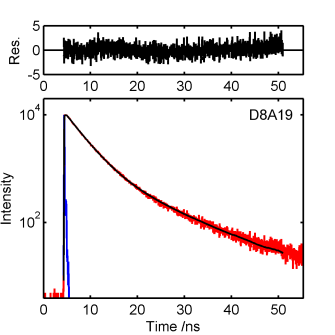 |
| 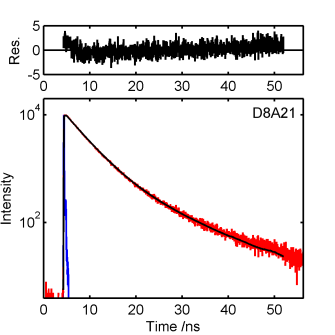 | 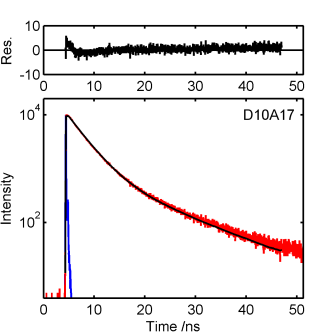 | 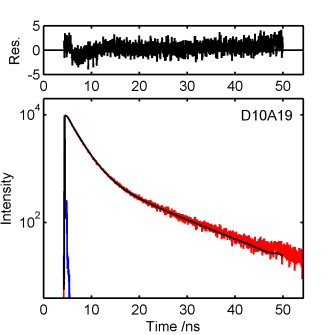 | 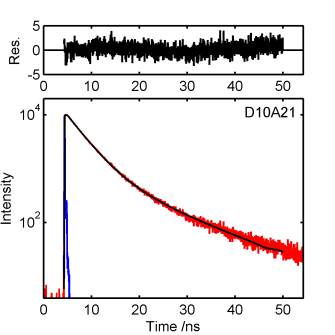 |
| 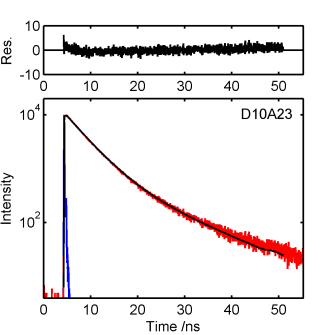 | 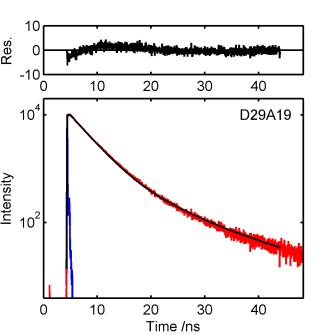 | 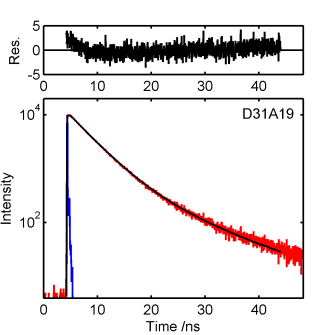 | 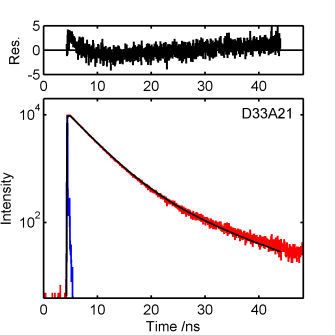 |
|  | 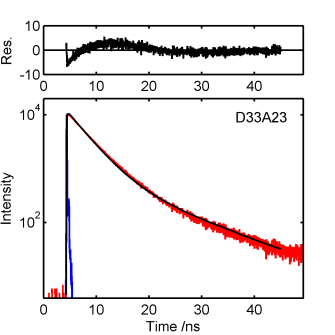 | 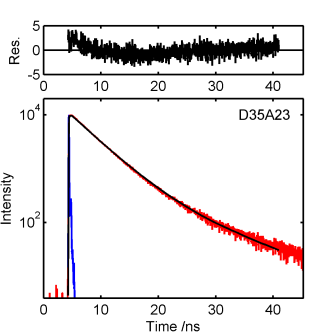 |  |

Supplementary Figure 9. Global fit of decays of 0A bulge in demonstration study 2. Blue: Instrument Response Function (IRF); Red: Measured decay; Black: Simulated, convolved decay. Fits were obtained at kink parameter values: *v*_x_ = -0.5 Å, *v*_y_ = -1.0 Å, *v*_z_ = 3.3 Å, α+γ = 31°, β = 0° (Fig. 5d in paper).

| 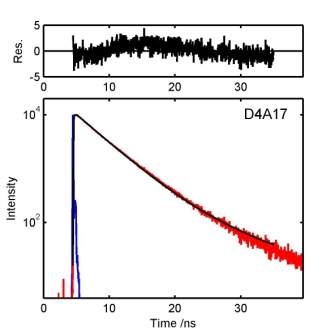 | 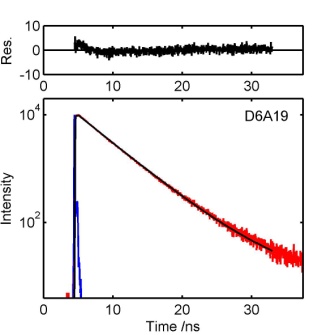 | 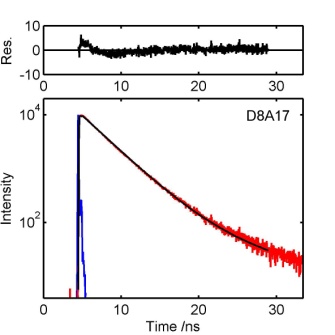 | 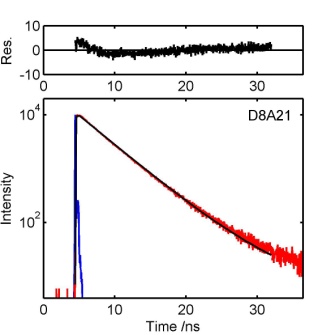 |
| --- | --- | --- | --- |
| 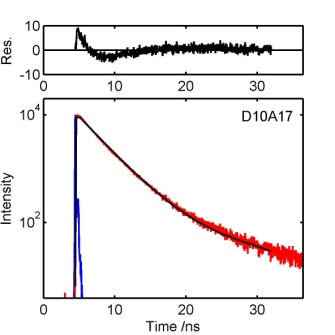 | 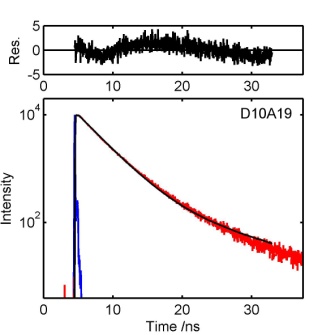 | 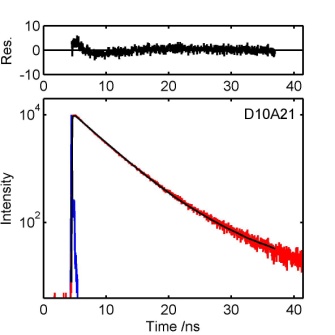 | 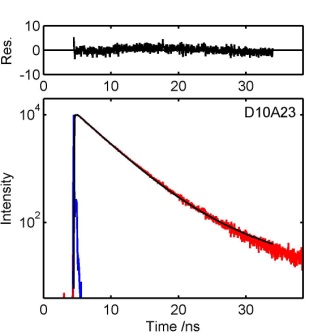 |
| 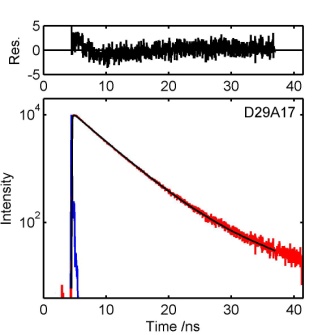 | 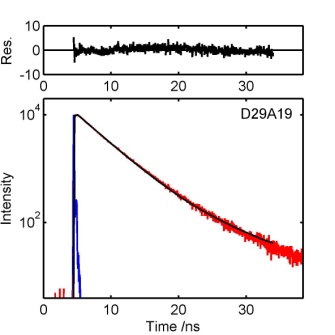 | 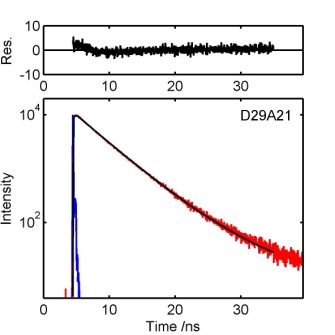 | 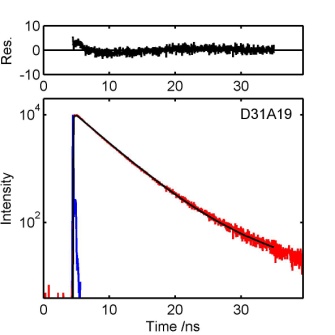 |
| 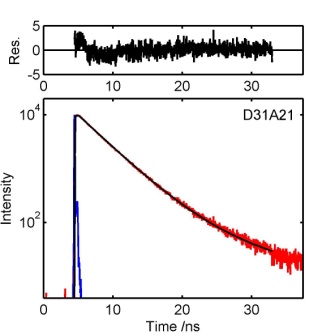 | 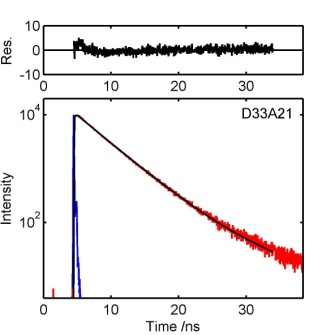 | 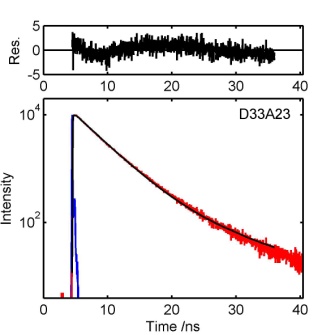 | 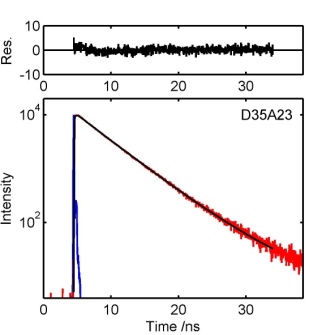 |

Supplementary Figure 10. Global fit of 3A bulge decays in demonstration study 2. Blue: Instrument Response Function (IRF); Red: Measured decay; Black: Simulated, convolved decay. Fits were obtained with kink parameter values: *v*_x_ = 7.4 Å, *v*_y_ = -0.5 Å, *v*_z_ = 5.5 Å, α = 101°, β = 57°, γ = -43°.


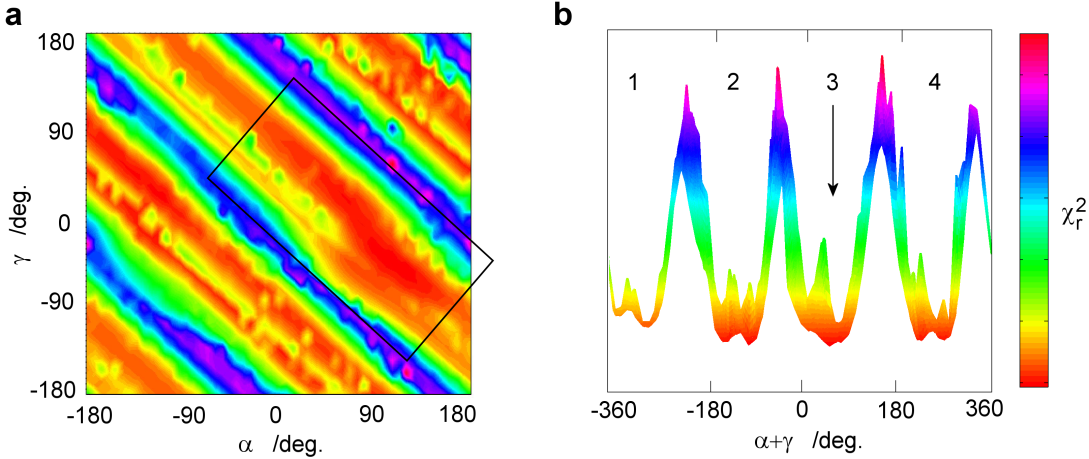


Supplementary Figure 11. (a) Full α,γ chi-square surface of 3A bulge model system. (b) Sideview of surface in (a) looking from lower right corner to upper left corner. The chi-square oscillates with a period of α+γ = 180° as is also observed for the 0A bulge model system (Fig. 5d in paper). These oscillations are caused by the symmetric properties of the two DNA helices separated by the kink. We interpret trough 3 (α+γ ≈ 55°) as the true global minimum based on the assumption that the DNA bending follows the right handed helical twist of the two helices. Constructing all other kink parameter chi-square surfaces (Fig. 5e in paper) were done with the values of α and γ optimized within the intervals marked by the black rectangle in (a). Color bar: 1.85:13 for both (a) and (b).


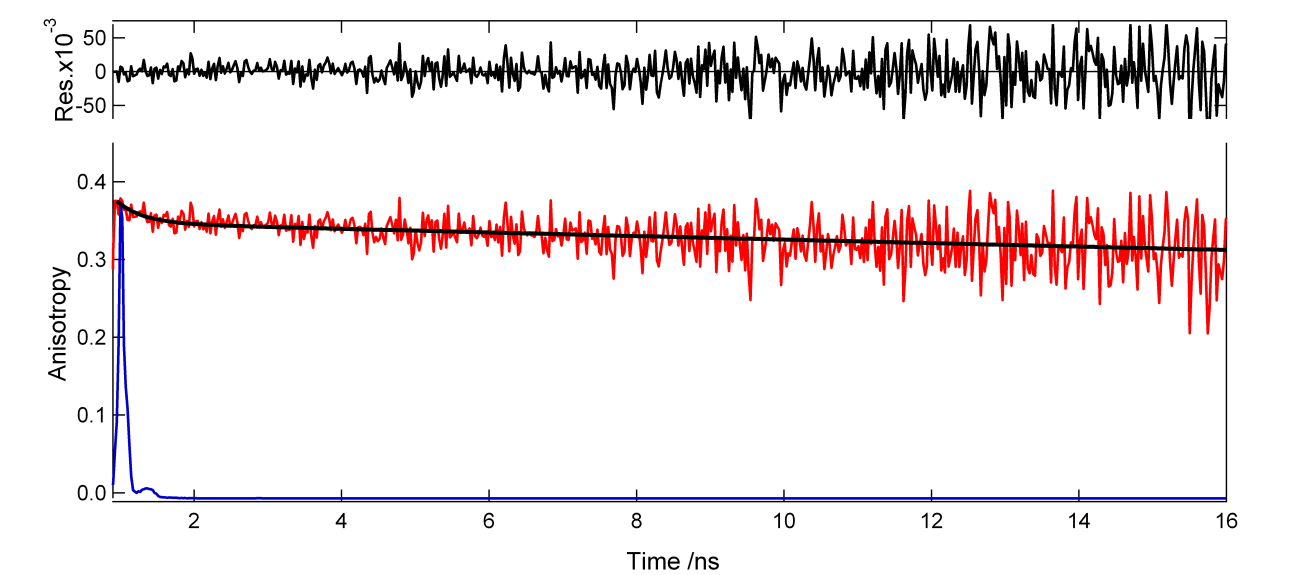


Supplementary Figure 12. Time-resolved fluorescence anisotropy of tC^O^ in double-stranded DNA in high viscosity phosphate buffer solution (55% sucrose, 0.1 M Na^+^, *T* = 296 K). In high viscosity solution, the rotational correlation times affecting the anisotropy decay in the measurable timescales are those due to DNA torsional motions with a long rotational correlation of *τ*_2_, and the fast internal fluorophore motions with a rotational correlation time of *τ_F_* while all other rotational motions can be assumed as infinite. In this regime the decay is fitted to

$$r\left( t \right)=r_{0}\left[ \alpha e^{-\frac{t}{\tau_{F}}}+\left( 1-\alpha\right) \right]e^{-\frac{t}{\tau_{2}}}$$

Here, *r*_0_ is the fundamental anisotropy of the probe, 0.375,([2](#_ENREF_2)) *α* is a parameter in between 0 and 1 representing the internal rotational flexibility of the probe. The measured time-resolved anisotropy decay (red) was fitted using α, *τ_F_* and *τ*_2_ as fitting parameters:

| Sample | *r_0_* | *α* | *τ_F_* /ns | *τ*_2_ /ns | *R*^2^ |
| --- | --- | --- | --- | --- | --- |
| 180bp, high visc. | 0.375 | 0.075 | 0.35 | 143 | 1.39 |

# SUPPLEMENTARY TABLES

Supplementary Table 1. Atomic coordinates of tC, tC^O^ and tC_nitro_ in the nucleobase coordinate frames defined in Supplementary Figure 1 (the bases positioned in strand I). “Dipole center” and “dipole end” are used to define the position and orientation of the transition dipole vectors. The dipole end is the end point of the unit vector going from the dipole center and tilted an angle *δ* relative to the molecular long axis and is calculated as: (*x*,*y*,*z*) = (Center(*x*,*y*,*z*) + (cos(*δ*+*L*), -sin(*δ*+*L*), 0)), where *L* = 0^0^, *L* = 5^0^ and *L* = 3^0^ for tC^O^, tC and tC_nitro_, respectively (Supplementary Figure 1). Atomic coordinates are based on the DFT B3LYP/6-31G** optimized geometries. The ground-state structures of tC and tC_nitro_ are degenerate, corresponding to two geometries folded along the middle sulphur/oxygen-nitrogen axis([6](#_ENREF_6)), however, only the coordinates of the average, planar geometry of tC and tC_nitro_ are shown. Because of the flexibility of the tricyclic frameworks, the out-of-plane bending was set as a variable in the analysis as described in the paper.

| tC^O^ | | | |  | tC | | |  | tC_nitro_ | | |
| --- | --- | --- | --- | --- | --- | --- | --- | --- | --- | --- | --- |
| Atom | x | y | z |  | x | y | z |  | x | y | z |
| N1 | 0.000 | -1.387 | 0.000 |  | 0.000 | -1.371 | 0.000 |  | 0.000 | -1.360 | 0.000 |
| C2 | -1.206 | -0.726 | 0.000 |  | -1.202 | -0.736 | 0.000 |  | -1.220 | -0.715 | 0.000 |
| N3 | -1.179 | 0.697 | 0.000 |  | -1.181 | 0.691 | 0.000 |  | -1.186 | 0.711 | 0.000 |
| C4 | 0.000 | 1.387 | 0.000 |  | 0.000 | 1.383 | 0.000 |  | 0.000 | 1.395 | 0.000 |
| O5/S5 | 2.371 | 1.438 | 0.000 |  | 2.716 | 1.683 | 0.000 |  | 2.718 | 1.671 | 0.000 |
| C6 | 4.737 | 1.416 | 0.000 |  | 5.283 | 0.774 | 0.000 |  | 5.266 | 0.736 | 0.000 |
| C7 | 5.947 | 0.729 | 0.000 |  | 6.303 | -0.175 | 0.000 |  | 6.258 | -0.241 | 0.000 |
| C8 | 5.956 | -0.659 | 0.000 |  | 5.980 | -1.531 | 0.000 |  | 5.946 | -1.598 | 0.000 |
| C9 | 4.758 | -1.363 | 0.000 |  | 4.646 | -1.927 | 0.000 |  | 4.610 | -1.970 | 0.000 |
| N10 | 2.316 | -1.357 | 0.000 |  | 2.289 | -1.429 | 0.000 |  | 2.268 | -1.440 | 0.000 |
| C | 1.128 | -0.690 | 0.000 |  | 1.138 | -0.687 | 0.000 |  | 1.118 | -0.682 | 0.000 |
| C | 1.180 | 0.737 | 0.000 |  | 1.196 | 0.747 | 0.000 |  | 1.190 | 0.747 | 0.000 |
| C | 3.545 | 0.713 | 0.000 |  | 3.940 | 0.387 | 0.000 |  | 3.926 | 0.362 | 0.000 |
| C | 3.543 | -0.686 | 0.000 |  | 3.613 | -0.980 | 0.000 |  | 3.586 | -1.007 | 0.000 |
| O | -2.272 | -1.314 | 0.000 |  | -2.283 | -1.305 | 0.000 |  | -2.302 | -1.278 | 0.000 |
| N |  |  |  |  |  |  |  |  | 7.661 | 0.173 | 0.000 |
| O |  |  |  |  |  |  |  |  | 7.901 | 1.382 | 0.000 |
| O |  |  |  |  |  |  |  |  | 8.518 | -0.710 | 0.000 |
|  |  |  |  |  |  |  |  |  |  |  |  |
| Dipole:  Center | 2.360 | 0.000 | 0.000 |  | 2.450 | -0.176 | 0.000 |  | 2.450 | -0.176 | 0.000 |
| End^†^ | 3.199 | -0.545 | 0.000 |  | 3.216 | -0.819 | 0.000 |  | 3.364 | 0.231 | 0.000 |

**Supplementary Table 2.** Atomic coordinates of tC, tC^O^ and tC_nitro_ in the base-pair coordinate frame defined in **Fig. 1b left** of the paper. Dipole center and dipole end define the position and orientation of the transition dipoles. All base-pair coordinates in the base-pair reference frames were constructed from the individual base coordinate frames using the base-pair parameter values corresponding to flat base-pairs([7](#_ENREF_7)):

propeller = 0^0^, opening = 0^0^, buckle = 0^0^, stagger = 0 Å, stretch = 5.45 Å, shear = 0 Å

The above definitions are all for the base analogues positioned in strand I and the complementary base in strand II as defined in the CEHS scheme.([8](#_ENREF_8)) Base analogues positioned in strand II have identical coordinates as for strand I except all *y* values are multiplied by -1.

|  | tC^O^ | | |  | tC | | |  | tC_nitro_ | | |
| --- | --- | --- | --- | --- | --- | --- | --- | --- | --- | --- | --- |
| Atom | x | Y | z |  | x | y | z |  | x | y | z |
| N1 | -0.465 | 2.154 | 0.000 |  | -0.465 | 2.152 | 0.000 |  | -0.465 | 2.157 | 0.000 |
| C2 | -1.545 | 2.990 | 0.000 |  | -1.542 | 2.981 | 0.000 |  | -1.558 | 2.999 | 0.000 |
| N3 | -1.279 | 4.388 | 0.000 |  | -1.281 | 4.384 | 0.000 |  | -1.284 | 4.399 | 0.000 |
| C4 | 0.000 | 4.869 | 0.000 |  | 0.000 | 4.867 | 0.000 |  | 0.000 | 4.873 | 0.000 |
| O5/S5 | 2.346 | 4.519 | 0.000 |  | 2.728 | 4.704 | 0.000 |  | 2.726 | 4.687 | 0.000 |
| C6 | 4.674 | 4.098 | 0.000 |  | 5.104 | 3.375 | 0.000 |  | 5.080 | 3.336 | 0.000 |
| C7 | 5.751 | 3.217 | 0.000 |  | 5.950 | 2.267 | 0.000 |  | 5.893 | 2.205 | 0.000 |
| C8 | 5.525 | 1.847 | 0.000 |  | 5.402 | 0.985 | 0.000 |  | 5.356 | 0.920 | 0.000 |
| C9 | 4.226 | 1.356 | 0.000 |  | 4.021 | 0.820 | 0.000 |  | 3.977 | 0.779 | 0.000 |
| N10 | 1.820 | 1.774 | 0.000 |  | 1.782 | 1.709 | 0.000 |  | 1.758 | 1.696 | 0.000 |
| C | 0.761 | 2.631 | 0.000 |  | 0.772 | 2.635 | 0.000 |  | 0.752 | 2.637 | 0.000 |
| C | 1.053 | 4.029 | 0.000 |  | 1.071 | 4.038 | 0.000 |  | 1.064 | 4.034 | 0.000 |
| C | 3.380 | 3.606 | 0.000 |  | 3.715 | 3.220 | 0.000 |  | 3.696 | 3.193 | 0.000 |
| C | 3.142 | 2.228 | 0.000 |  | 3.162 | 1.928 | 0.000 |  | 3.130 | 1.901 | 0.000 |
| O | -2.695 | 2.590 | 0.000 |  | -2.704 | 2.603 | 0.000 |  | -2.720 | 2.626 | 0.000 |
| N |  |  |  |  |  |  |  |  | 7.345 | 2.377 | 0.000 |
| O |  |  |  |  |  |  |  |  | 7.786 | 3.528 | 0.000 |
| O |  |  |  |  |  |  |  |  | 8.041 | 1.362 | 0.000 |
|  |  |  |  |  |  |  |  |  |  |  |  |
| Dipole:  Center | 2.092 | 3.104 | 0.000 |  | 2.150 | 2.911 | 0.000 |  | 2.150 | 2.911 | 0.000 |
| End | 2.827 | 2.425 | 0.000 |  | 2.798 | 2.154 | 0.000 |  | 3.119 | 3.158 | 0.000 |

Supplementary Table 3. D­NA sequences in demonstration study 1. X = tC^O^. Y = tC_nitro_.

| **Name** | **Sequence** |
| --- | --- |
| D7 | 5’-CGA TCA **X**AC ACA AGG ACG AGG ATA AGG AGG AGG |
| D9 | 5’-CGA TCA CA**X** ACA AGG ACG AGG ATA AGG AGG AGG |
| D11 | 5’-CGA TCA CAC A**X**A AGG ACG AGG ATA AGG AGG AGG |
| A14 | 5’-CCT CCT CCT TAT CCT CGT C**Y**T TGT GTG TGA TCG |
| A15 | 5’-CCT CCT CCT TAT CCT CGT **Y**CT TGT GTG TGA TCG |
| A20 | 5’-CCT CCT CCT TAT C**Y**T CGT CCT TGT GTG TGA TCG |
| A21 | 5’-CCT CCT CCT TAT **Y**CT CGT CCT TGT GTG TGA TCG |

Supplementary Table 4. Parameter values in demonstration study 1.

| Parameter | Value |
| --- | --- |
| *J* / M^-1^cm^-1^nm^4^ | 1.3e14 |
| Φ_D_ | 0.23 (reference ([2](#_ENREF_2))) |
| *τ*_D_ /ns | 4.32, 4.35, 4.55 |
| *a* | 0.992−1 |
| *b* | 0 |
| *η* | 1.4 |

Supplementary Table 5. DNA sequences in demonstration study 2.

| **Name** | **Donor sequences (X = tC^O^): 0A-bulge** |
| --- | --- |
| D4 | 5’-CCA **X**AC ACA CAC GTG AGA GAG AGA CGT ACA CAC ACT CC |
| D6 | 5’-CCA CA**X** ACA CAC GTG AGA GAG AGA CGT ACA CAC ACT CC |
| D8 | 5’-CCA CAC A**X**A CAC GTG AGA GAG AGA CGT ACA CAC ACT CC |
| D10 | 5’-CCA CAC ACA **X**AC GTG AGA GAG AGA CGT ACA CAC ACT CC |
| D29 | 5’-CCA CAC ACA CAC GTG AGA GAG AGA CGT A**X**A CAC ACT CC |
| D31 | 5’-CCA CAC ACA CAC GTG AGA GAG AGA CGT ACA **X**AC ACT CC |
| D33 | 5’-CCA CAC ACA CAC GTG AGA GAG AGA CGT ACA CA**X** ACT CC |
| D35 | 5’-CCA CAC ACA CAC GTG AGA GAG AGA CGT ACA CAC A**X**T CC |
|  | **Donor sequences (X = tC^O^): 3A-bulge** |
| D4 | 5’-CCA **X**AC ACA CAC G***AAA***TG AGA GAG AGA CGT ACA CAC ACT CC |
| D6 | 5’-CCA CA**X** ACA CAC G***AAA***TG AGA GAG AGA CGT ACA CAC ACT CC |
| D8 | 5’-CCA CAC A**X**A CAC G***AAA***TG AGA GAG AGA CGT ACA CAC ACT CC |
| D10 | 5’-CCA CAC ACA **X**AC G***AAA***TG AGA GAG AGA CGT ACA CAC ACT CC |
| D29 | 5’-CCA CAC ACA CAC GTG AGA GAG AGA CG***AAA***T A**X**A CAC ACT CC |
| D31 | 5’-CCA CAC ACA CAC GTG AGA GAG AGA CG***AAA***T ACA **X**AC ACT CC |
| D33 | 5’-CCA CAC ACA CAC GTG AGA GAG AGA CG***AAA***T ACA CA**X** ACT CC |
| D35 | 5’-CCA CAC ACA CAC GTG AGA GAG AGA CG***AAA***T ACA CAC A**X**T CC |
|  | **Non-modified strands** |
|  | 5’-CCA CAC ACA CAC GTG AGA GAG AGA CGT ACA CAC ACT CC |
|  | 5’-GGA GTG TGT GTA CGT CTC TCT CTC ACG TGT GTG TGT GG |
|  | **Acceptor sequences (Y = tC_nitro_)** |
| A15 | 5’-GGA GTG TGT GTA CGT CTC TCT CT**Y** ACG TGT GTG TGT GG |
| A17 | 5’-GGA GTG TGT GTA CGT CTC TCT **Y**TC ACG TGT GTG TGT GG |
| A19 | 5’-GGA GTG TGT GTA CGT CTC T**Y**T CTC ACG TGT GTG TGT GG |
| A21 | 5’-GGA GTG TGT GTA CGT CT**Y** TCT CTC ACG TGT GTG TGT GG |
| A23 | 5’-GGA GTG TGT GTA CGT **Y**TC TCT CTC ACG TGT GTG TGT GG |

Supplementary Table 6. Double stranded donor reference decays of demonstration study 2 fitted to a double-exponential decay:

$$I(t)=I_{0}\left( \alpha_{1}e^{-t/\tau_{1}}+\alpha_{2}e^{-t/\tau_{2}} \right)$$

All decays were fitted in one global step with the value of *τ*_2_ set to be equal for all samples, *τ*_1_ set to be equal for the same position in the strand (e.g. 0A-D35 and 3A-D35) while *α*_1_ and *α*_2_ = 1 - *α*_1_ were allowed to vary in between samples. The long lifetime of *τ*_2_ = 12.01 ns is the result of an unidentified impurity present in all samples of demonstration study 2, while *τ*_1_ is the tC^O^ donor reference lifetime.

| **0A** | ***α*_1_** | ***α*_2_** | ***τ*_1_** | ***τ*_2_** | $\boldsymbol{\chi}^{\boldsymbol{2}}$ |
| --- | --- | --- | --- | --- | --- |
| D4 | 0.96 | 0.045 | 4.21 | 12.01 | 1.11 |
| D6 | 0.95 | 0.054 | 4.24 | 12.01 | 1.13 |
| D8 | 0.94 | 0.056 | 4.17 | 12.01 | 1.04 |
| D10 | 0.95 | 0.054 | 4.16 | 12.01 | 1.03 |
| D29 | 0.96 | 0.041 | 4.28 | 12.01 | 1.10 |
| D31 | 0.96 | 0.036 | 4.25 | 12.01 | 1.03 |
| D33 | 0.96 | 0.040 | 4.32 | 12.01 | 1.10 |
| D35 | 0.97 | 0.034 | 4.60 | 12.01 | 0.99 |
| **3A** | ***α*_1_** | ***α*_2_** | ***τ*_1_** | ***τ*_2_** | $\boldsymbol{\chi}^{\boldsymbol{2}}$ |
| D4 | 0.99 | 0.013 | 4.21 | 12.01 | 1.15 |
| D6 | 0.99 | 0.013 | 4.24 | 12.01 | 1.08 |
| D8 | 0.99 | 0.011 | 4.17 | 12.01 | 1.15 |
| D10 | 0.98 | 0.016 | 4.16 | 12.01 | 1.16 |
| D29 | 0.98 | 0.017 | 4.28 | 12.01 | 1.06 |
| D31 | 0.99 | 0.015 | 4.25 | 12.01 | 1.02 |
| D33 | 0.98 | 0.017 | 4.32 | 12.01 | 1.10 |
| D35 | 0.99 | 0.012 | 4.60 | 12.01 | 1.05 |
| Global |  |  |  |  | 1.08 |

Supplementary Table 7. Fit of tC^O^ donor decays of demonstration study 2 in presence of FRET using a double-exponential decay. The value of *τ*_2_ were constrained to 12.01 ns for all samples while *τ*_1_, *α*_1_, and *α*_2_ = 1- *α*_1_ were optimized for each sample. The values of *α*_1_ from these fits were used as the parameter *b* in the decay model when analyzing the decays during the FRET analysis.

| **0A** | ***α*_1_** | ***α*_2_** | ***τ*_1_** | ***τ*_2_** | $\boldsymbol{\chi}^{\boldsymbol{2}}$ |
| --- | --- | --- | --- | --- | --- |
| D4A15 | 0.966 | 0.034 | 3.61 | 12.01 | 1.15 |
| D4A17 | 0.962 | 0.038 | 3.98 | 12.01 | 1.20 |
| D6A15 | 0.975 | 0.025 | 2.53 | 12.01 | 1.20 |
| D6A17 | 0.955 | 0.045 | 3.60 | 12.01 | 1.11 |
| D6A19 | 0.950 | 0.050 | 3.98 | 12.01 | 1.08 |
| D8A15 | 0.964 | 0.036 | 3.12 | 12.01 | 1.20 |
| D8A17 | 0.973 | 0.027 | 2.51 | 12.01 | 1.29 |
| D8A19 | 0.954 | 0.046 | 3.60 | 12.01 | 1.12 |
| D8A21 | 0.950 | 0.050 | 3.98 | 12.01 | 1.05 |
| D10A17 | 0.965 | 0.035 | 3.17 | 12.01 | 1.13 |
| D10A19 | 0.976 | 0.024 | 2.50 | 12.01 | 1.23 |
| D10A21 | 0.957 | 0.043 | 3.61 | 12.01 | 1.11 |
| D10A23 | 0.950 | 0.050 | 3.99 | 12.01 | 1.10 |
| D29A19 | 0.967 | 0.033 | 3.83 | 12.01 | 1.02 |
| D31A19 | 0.966 | 0.034 | 3.93 | 12.01 | 1.04 |
| D33A21 | 0.965 | 0.035 | 3.89 | 12.01 | 1.13 |
| D33A23 | 0.967 | 0.033 | 3.78 | 12.01 | 1.06 |
| D35A23 | 0.971 | 0.029 | 4.17 | 12.01 | 1.09 |
| Global |  |  |  |  | 1.13 |
| **3A** | ***α*1** | ***α*2** | ***τ*1** | ***τ*2** | $\boldsymbol{\chi}^{\boldsymbol{2}}$ |
| D4A17 | 0.99 | 0.014 | 4.18 | 12.01 | 1.27 |
| D6A19 | 0.99 | 0.011 | 4.02 | 12.01 | 1.19 |
| D8A17 | 0.99 | 0.007 | 3.12 | 12.01 | 1.31 |
| D8A21 | 0.99 | 0.009 | 3.79 | 12.01 | 1.13 |
| D10A17 | 0.99 | 0.009 | 2.90 | 12.01 | 2.18 |
| D10A19 | 0.99 | 0.013 | 3.29 | 12.01 | 1.74 |
| D10A21 | 0.98 | 0.019 | 4.04 | 12.01 | 1.63 |
| D10A23 | 0.99 | 0.014 | 3.88 | 12.01 | 1.32 |
| D29A17 | 0.99 | 0.018 | 4.15 | 12.01 | 1.14 |
| D29A19 | 0.99 | 0.015 | 3.97 | 12.01 | 1.19 |
| D29A21 | 0.99 | 0.013 | 4.05 | 12.01 | 1.13 |
| D31A19 | 0.98 | 0.017 | 3.94 | 12.01 | 1.38 |
| D31A21 | 0.99 | 0.010 | 3.51 | 12.01 | 1.27 |
| D33A21 | 0.99 | 0.012 | 4.01 | 12.01 | 1.17 |
| D33A23 | 0.98 | 0.016 | 3.86 | 12.01 | 1.57 |
| D35A23 | 0.99 | 0.010 | 4.43 | 12.01 | 1.17 |
| Global |  |  |  |  | 1.36 |

Supplementary Table 8. Parameter values in demonstration study 2 for both the 0A and 3A structures.

| Parameter | Value |
| --- | --- |
| *J* / M^-1^cm^-1^nm^4^ | 1.3e14 |
| Φ_D_ | 0.23 for AA neighbours([2](#_ENREF_2))  0.27 for AT neighbours([2](#_ENREF_2)) |
| *τ*_D_ /ns | *τ*_1_ from Supplementary Table 6 |
| *A* | 0.996 |
| *B* | *α*_1_ from Supplementary Table 7 |
| *η* | 1.4 |

Supplementary Table 9. Fitted kink parameter values of the 3A bulge in demonstration study 2 with (+) and without (-) geometrical constraints. The minimum without constraints is in violence with constraint #2: maximum distance between neighbouring bases in the same strand.

| Parameter | + | - |
| --- | --- | --- |
| *v_x_* | 7.4 Å | 8.5 Å |
| v_y_ | -0.5 Å | -1.8 Å |
| *v_z_* | 5.5 Å | 5.01 Å |
| *α* | 101° | 101° |
| *β* | 57° | 64° |
| *γ* | -43° | -43° |
| $\chi_{\text{r}}^{2}$ | 1.85 | 1.83 |

Supplementary Table 10. Base-pair step parameters of regular A-form and B-form helices used by FRETmatrix. The sequence dependent parameters shown in the top are from the calf thymus fiber models of Arnott & co-workers([9](#_ENREF_9),[10](#_ENREF_10)) as implemented in w3DNA ([11](#_ENREF_11)). Average A-DNA and B-DNA parameters are from Olson *et al*. ([12](#_ENREF_12)).

| Step (5’-3’) | Shift /Å | Slide /Å | Rise /Å | Tilt /deg. | Roll /deg. | Twist /deg. |
| --- | --- | --- | --- | --- | --- | --- |
| AA | 0 | 0.45 | 3.36 | 0 | 1.71 | 35.96 |
| AT | 0 | 0.44 | 3.35 | 0 | 1.71 | 35.67 |
| AC | 0 | 0.49 | 3.36 | 0 | 1.71 | 36.83 |
| AG | 0 | 0.42 | 3.35 | 0 | 1.71 | 34.79 |
| TA | 0 | 0.47 | 3.36 | 0 | 1.71 | 36.26 |
| TT | 0 | 0.45 | 3.36 | 0 | 1.71 | 35.96 |
| TC | 0 | 0.50 | 3.37 | 0 | 1.71 | 37.13 |
| TG | 0 | 0.44 | 3.35 | 0 | 1.71 | 35.09 |
| CA | 0 | 0.44 | 3.35 | 0 | 1.71 | 35.10 |
| CT | 0 | 0.42 | 3.35 | 0 | 1.71 | 34.79 |
| CC | 0 | 0.47 | 3.36 | 0 | 1.71 | 35.96 |
| CG | 0 | 0.41 | 3.34 | 0 | 1.71 | 33.92 |
| GA | 0 | 0.50 | 3.37 | 0 | 1.71 | 37.13 |
| GT | 0 | 0.49 | 3.36 | 0 | 1.71 | 36.83 |
| GC | 0 | 0.54 | 3.38 | 0 | 1.71 | 38.00 |
| GG | 0 | 0.47 | 3.36 | 0 | 1.71 | 35.96 |
|  |  |  |  |  |  |  |
| Average: |  |  |  |  |  |  |
| B-DNA | -0.02 | 0.23 | 3.32 | -0.1 | 0.60 | 36.00 |
| A-DNA | 0 | -1.53 | 3.32 | 0.1 | 8.00 | 31.10 |

# Supplementary Note 1. Simulating FRET using three dimensional dipole vector representations

When the global coordinate matrix of a structure has been constructed the coordinates defining the transition dipoles of donor and acceptor in the global coordinate frame are extracted. We denote these by

$$Donor center=\left( \begin{matrix} x_{1} \\ y_{1} \\ z_{1} \end{matrix} \right) , Donor end=\left( \begin{matrix} x_{1,end} \\ y_{1,end} \\ z_{1,end} \end{matrix} \right)$$

$Acceptor center=\left( \begin{matrix} x_{2} \\ y_{2} \\ z_{2} \end{matrix} \right)$ , $Acceptor end=\left( \begin{matrix} x_{2,end} \\ y_{2,end} \\ z_{2,end} \end{matrix} \right)$

The normalized dipole unit vectors in the global coordinate frame of the nucleic acid are:

$$\vec{e_{1}}=\left( \begin{matrix} x_{1,end}-x_{1} \\ y_{1,end}-y_{1} \\ z_{1,end}-z_{1} \end{matrix} \right) , \vec{e_{2}}=\left( \begin{matrix} x_{2,end}-x_{2} \\ y_{2,end}-y_{2} \\ z_{2,end}-z_{2} \end{matrix} \right)$$

The FRET efficiency is then calculated as

$$E=\frac{R_{0}^{6}}{R_{0}^{6}+R^{6}}$$

where *R*_0_ is the critical Förster distance and *R* is the distance between the dipoles:

$$R=\sqrt{\left( x_{2}-x_{1} \right)^{2}+\left( y_{2}-y_{1} \right)^{2}+\left( z_{2}-z_{1} \right)^{2}}$$

The critical Förster distance is given by

$$R_{0}=0.211\left( \frac{\kappa^{2}\Phi_{D}J\left( \lambda\right)}{\eta^{-4}} \right)^{\frac{1}{6}}$$

where *κ*^2^ is the orientation factor, Φ_D_ is the donor fluorescence quantum yield is absence of acceptor, *η* is the refractive index, *J* is the spectral overlap integral between donor emission and acceptor absorption calculated as

$$J\equiv\int_{0}^{\infty} \varepsilon_{A}\left( \lambda\right)\lambda^{4}F_{D}\left( \lambda\right)d\lambda$$

Here *ε*_A_ is the acceptor molar absorptivity spectrum, *λ* is the wavelength, and *F*_D_ is the normalized donor emission spectrum. The orientation factor is given by (see Fig. 1d in paper)

$$\kappa=\vec{e_{1}}\cdot\vec{e_{2}}-3\left( \vec{e_{1}}\cdot\vec{e_{12}} \right)\left( \vec{e_{12}}\cdot\vec{e_{2}} \right)$$

where the unit vector between the two dipole centres is calculated as

$$\vec{e_{12}}=\frac{1}{R}\left( \begin{matrix} x_{2}-x_{1} \\ y_{2}-y_{1} \\ z_{2}-z_{1} \end{matrix} \right)$$

#### Simulating FRET between dipole vector distributions

If *N* donor and *N* acceptor dipoles are sampled in the simulation, the *κ*^2^ is calculated between every pair combination in the two distributions (*N*^2^ pairs in total) and the average *κ*^2^ is then evaluated as

$\left\langle\kappa^{2} \right\rangle=\frac{\sum_{i}^{N^{2}} \kappa_{i}^{2}}{N^{2}}$

In the dynamic averaging regime (dipole reorientation much faster than energy transfer rate) the FRET efficiency is calculated as

$E=\frac{1}{1+C^{-1}\times\frac{R^{6}}{\left\langle\kappa^{2} \right\rangle}}$

where $C={0.211}^{6}\left( \frac{\Phi_{D}J\left( \lambda\right)}{\eta^{-4}} \right)$.

# Supplementary Note 2. Flow chart for simulating dipole dynamics

The simulation of base-base FRET including dipole distributions is divided into three steps:

1. Construct atomic coordinate matrix of structure and extract dipole vectors

2. Construct dipole vector distributions

3. Simulate FRET between dipole distributions

Step 1 is described in the paper. Step 2 is divided into five steps:

2A. Define a potential energy function

2B. Construct Boltzmann probability density function, *P*(*x*), from potential

2C. Sample *P*(θ) and *P*(φ) in spherical coordinates

2D. Transform sampled spherical coordinates into Cartesian coordinates

2E. Align sampled distribution with dipole vector in local coordinate frame

2F. Position vector distribution into the global coordinate frame

Step 3 is further divided into four steps:

3A. Calculate <*κ*^2^> between sampled distributions of donor and acceptor

3B. Simulate intensity decays (or a steady-state FRET efficiency)

3C. Convolve simulated decay with measured IRF

3D. Evaluate simulated decays with measured: calculate *χ*^2^

When constructing chi-square surface the above procedure is repeated for a new set of parameters. All the resulting chi-square values are stored in a matrix for subsequent analysis and chi-square surface plots.

# Supplementary Note 3. FRETmatrix v1.0 user guide

FRETmatrix user guide

v1.0, Jan. 2012

Go to <https://sites.google.com/site/fretmatrix/> for updates and corrections.

Contact: [spreus@nano.ku.dk](mailto:spreus@nano.ku.dk) or [marcus.wilhelmsson@chalmers.se](mailto:marcus.wilhelmsson@chalmers.se)

# Requirements:

MATLAB 2009 or newer with the Bioinformatics Toolbox installed.

Some features in the analysis part require the MATLAB Signal Processing Toolbox, the Global Optimization Toolbox and the Statistics Toolbox 2010 or newer.

# Installation

The software does not need to be installed but is run directly from folder. Download the FRETmatrix zip file and unzip it to a local directory.

# Folders

‘Calls’: Contains the scripts and functions being called by FRETmatrix.

‘Inputs’: Contains input files for FRETmatrix supplied by the user.

‘Outputs’: Contains output files produced by FRETmatrix during and after a simulation or analysis.

Part I:

Simulating base-base FRET

# Open the FRETmatrix user interface

1. Open MATLAB
2. Make the FRETmatrix folder your current directory
3. In the command window type: FRETmatrix
4. Press enter. This will open the FRETmatrix user interface (Figure 13). The user interface can be used for the simulation of base-base FRET (steady state) in any nucleic acid geometry.


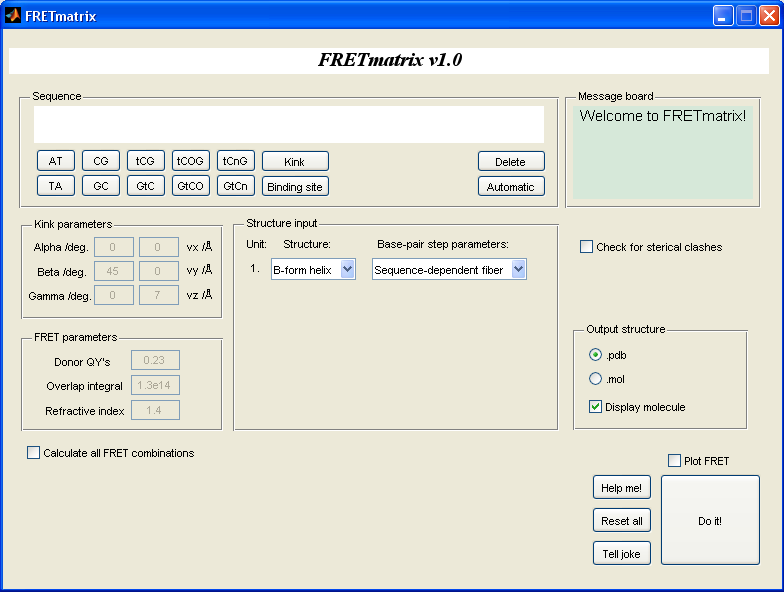


Figure 13. Start window of the FRETmatrix user interface.

# Panels and buttons

| **Sequence panel** | Here the sequence of the modeled structure is specified. Type in a sequence by pushing the buttons representing different base-pairs. ‘AT’ inserts an A positioned in strand I and a T and strand II, while ‘TA’ inserts a T positioned in strand I and an A in strand II, etc.. | |
| --- | --- | --- |
|  | **Kink button** | Inserts a kink after the last base-pair. A kink separates two structural units. The relative orientation and position of two neighbouring units are specified by the kink parameters (Figure 14). |
|  | **Binding site button** | The ‘Binding site’ button has the exact same function as ‘Kink’ in the program. |
|  | **Automatic button** | Generates an automated sequence of the active unit of the sequence (the last unit). Automatic has different meanings depending on the structure being simulated:   1. For A-form and B-form helices automatic puts in a default 10 bp sequence of pure CG base pairs. 2. For structures reconstructed from an input file the sequence is read directly from the file.   It is possible to have the sequence of one structural unit being automatic while other units are manually input. |
|  | **Delete button** | Deletes the last base-pair or kink of the sequence. |
| **Kink parameters panel** | Three Euler angles and three translation coordinates defining the relative orientation and position of two structural units. The angles are defined with respect to the coordinate frames of the last base-pair of the first unit and the first base-pair of the second unit (Figure 14). | |
| **FRET parameters panel** | Specifies the parameters needed to calculate the FRET characteristics between base probes in the structure. Different donor quantum yields can be specified for more than one donor. The overlap integral is in units of M^-1^cm^-1^nm^4^. | |
| **Structure input panel** | Here, the structural geometry of each unit in the sequence is specified. A new structural unit appears in this panel when a kink + the next base-pair is entered in the sequence (Figure 15). | |
|  | **Structure** | When choosing A-form or B-form helix the base-pair step parameters must be set to “sequence-dependent”, being the base-pair step parameters of Arnott’s fiber models as implemented in w3DNA, or “average”, being the average base-pair step parameters derived by Olson *et al*.[^8^](#_ENREF_8)^,^[^9^](#_ENREF_9)^,^[^11^](#_ENREF_11)  Choosing ‘Imported’ is used for reconstructing geometries defined in a pdb file. FRETmatrix requires an input file specifying the structural parameters necessary to rebuild the structure. Three different input files can be used: .par, .out, or .dat, all of which are output files produced by the structural analysis routine of the 3DNA software[^12^](#_ENREF_12). The .out file is also achievable using w3DNA[^10^](#_ENREF_10). Examples are provided in the inputs folder. |
| **Calculate all FRET combinations**  **checkbox** | Checking this box activates the associated panel (Figure 16) and automatically calculates FRET between all donor and acceptor positions in the sequence. This feature is for experiment design purposes. The number of FRET combinations can be limited by introducing constraints in the form of no. of base-pairs separating the donor and acceptor. A constrained site can also be inserted. Clicking this checkbox will only calculate FRET combinations in which the donor and acceptor are positioned on opposite sides of the constrained site. The results are exported to a txt file and an Excel file by pushing the checkbox ,’Export to Excel’. | |
| **Check for Sterical Clashes**  **checkbox** | This will display a warning if there are any sterical clashes between two bases in the structure. The program does this by calculating the distance between all atoms in the different base-pairs. A sterical clash is registered when two atoms are closer than two times the Van der Waal radius, i.e.: *r* < 2*vdw. | |
| **Output structure**  **panel** | The program generates a molecule file of the built structure for visual inspection purposes. Choose pdb or mol format as output. The pdb format is optimal for most purposes as there is no upper limit on the total number of atoms in the structure, which is the case for the mol format (>1000 atoms). | |
| **Plot FRET checkbox** | Checking this box will plot the resulting FRET efficiencies against the donor-acceptor distance. | |
| **Help me button** | Opens the html file called FRETmatrix_help.html located in the ‘Calls’ folder. | |
| **Reset all button** | Restarts the FRETmatrix user interface. | |

###
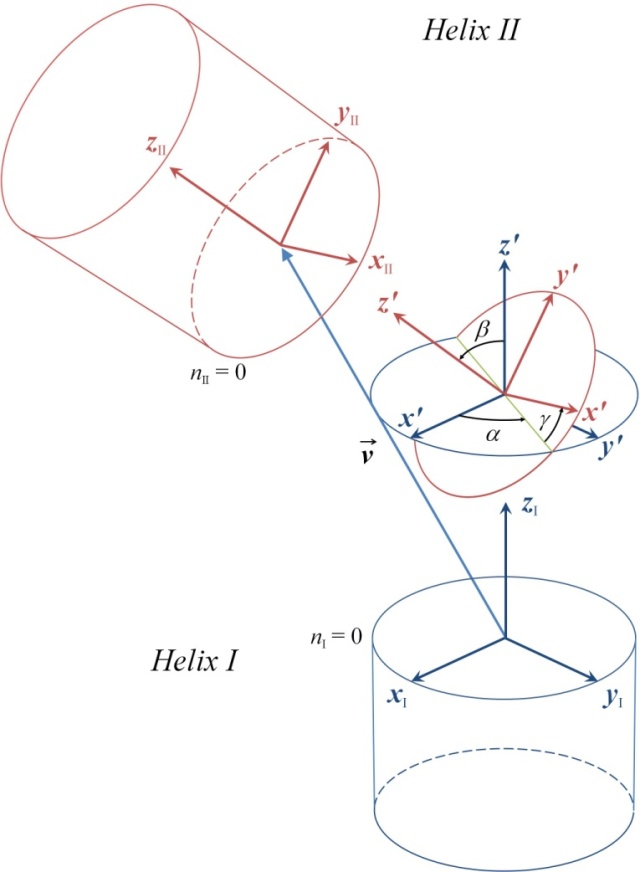


Figure 14. Parameters describing the relative position and orientation of two structural units separated by a kink. Alpha, beta, and gamma are three Euler angles describing the relative rotations according to the convention R_z_(α)R_x_(β)R_z_(γ). The translation vector, v, has three components: v_x_, v_y_, v_z_ defined with respect to the coordinate system of unit 1 (blue).

###
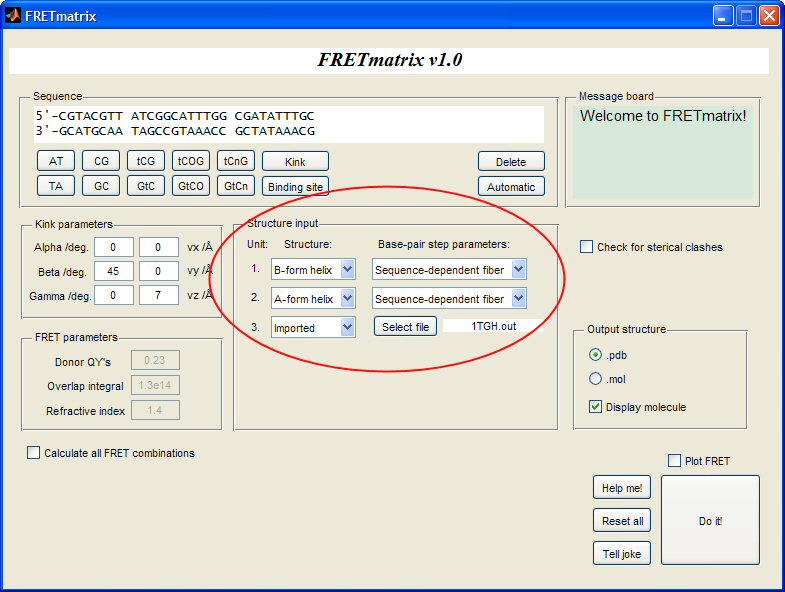


Figure 15. The structure input panel defines what structure is built from the input sequence. Here, two kinks and thus three structural units has been entered in the sequence.


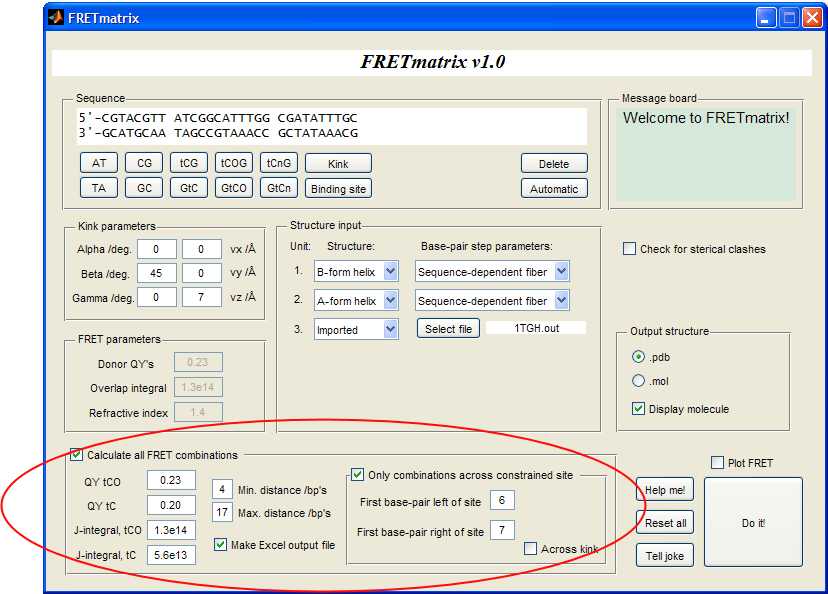


Figure 16. Activating the ‘Calculate all FRET combinations’ panel inserts FRET pairs at all possible combinations in the sequence and calculate the corresponding FRET characteristics. To narrow down the results list a number of constraints can be chosen, such as the D-A distance (in bps) and a constrained site. The results are exported to an Excel file located in the outputs folder.

### Quick example 1: Construct two B-DNA helices separated by a kink and simulate FRET between base probes positioned on opposite sides of the kink

1. Type in a sequence by pushing the base-pair buttons in sequential order. Insert donor base probes, e.g. ‘tCOG’, at any positions in the sequence.
2. After typing in the sequence of helix 1 (referred to as unit 1) press the ‘Kink’ button and start typing in the sequence of helix II. An extra structural unit appears in the ‘Structure input’ panel and the ‘Kink parameters’ panel becomes activated. In the sequence the two structural units are separated by a space
3. Insert acceptor base probes, e.g. ‘GtCn’, at any positions in the sequence of unit II. This activates the ‘FRET parameters’ panel.
4. In the ‘Kink parameters’ panel type in the six rigid body parameters describing the relative orientation and position of the two units (Figure 14). Using the default parameters, α = 0°, β = 45°, γ = 0°, will make a 45° bending angle between the two helices (unit 1 and unit 2).
5. In the ‘FRET parameters’ panel type in the parameters describing the FRET pair. See reference values in Sandin *et al.* and Preus *et al.*[^2^](#_ENREF_2)^,^[^3^](#_ENREF_3)^,^[^13^](#_ENREF_13).
6. Press ‘Do it’.

The program will construct a molecule file located in the ‘Outputs’ folder called mol.pdb. If the ‘Display molecule’ is checked the produced molecule file will be opened directly by the molviewer in MATLAB (). FRET will be simulated between all FRET pairs inserted in the sequence with the results being displayed in the MATLAB command window.


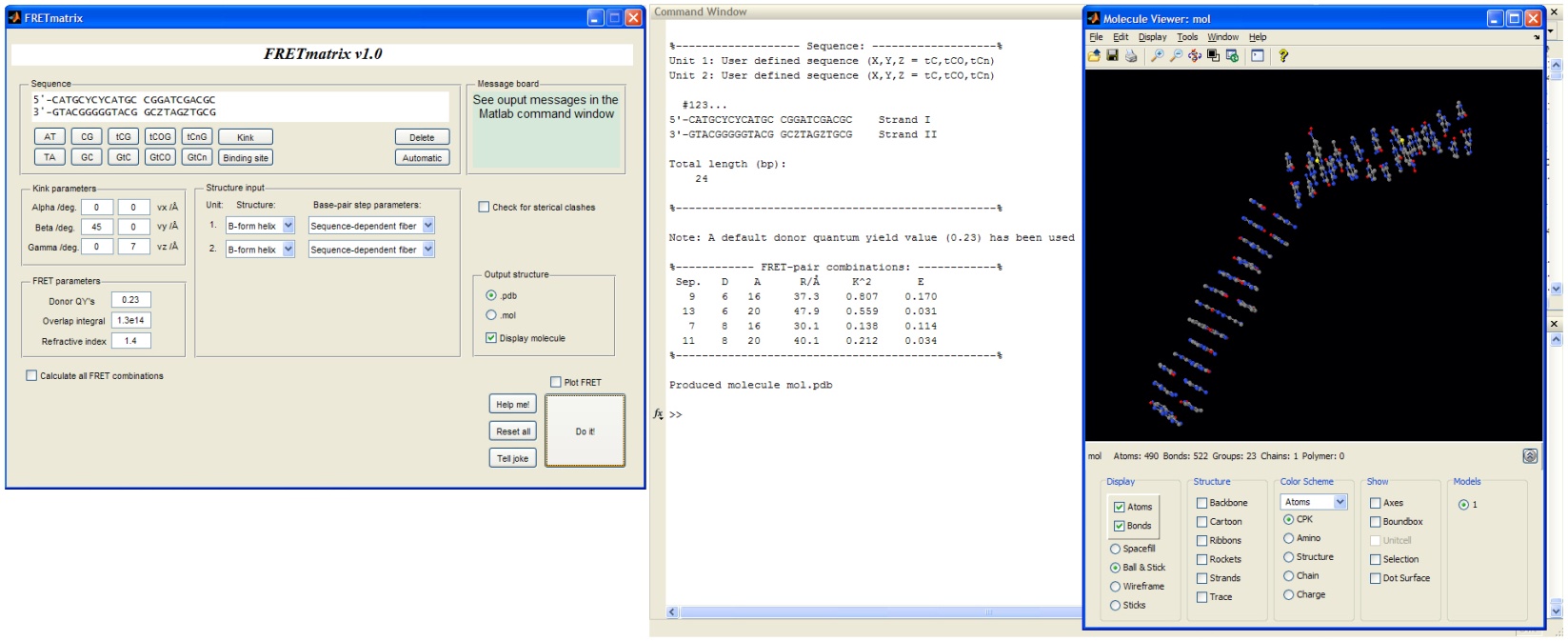


Figure 17. Screenshots from working example 1.

### Quick example 2: Reconstruct structure from pdb file and simulate FRET between all possible positions in the structure.

This example is useful for experiment design purposes.

1. Use w3DNA to analyze the pdb structure (<http://w3dna.rutgers.edu/>). Download the resulting output .out file. Examples can be found in the FRETmatrix/inputs folder.
2. Select ‘Automatic’ in the ‘Sequence’ panel.
3. In the ‘Structure input’ panel choose ‘Imported’ for unit 1. This will activate the ‘Select file’ button next to the unit in the ‘Structure input’ panel.
4. Press the ‘Select file’ button and localize the .out file and press ok. The ‘Inputs’ folder of FRETmatrix contains examples that can be used as well.
5. Press the ‘Calculate all’ checkbox. This activates the ‘Calculate all’ panel and will automatically combine all possible FRET combinations in the structure (also positions not occupied by a C in the sequence).
6. Press ‘Do it’

If the ‘Export to Excel’ is checked an Excel file is generated with the resulting FRET characteristics calculated for all FRET positions in the Structure. The Excel file contains the simulations for both tC and tC^O^ but in separate sheets. The file is saved in the FRETmatrix/outputs folder.

Note: For some structures, the output file of w3DNA cannot be used. In this case 3DNA must be used to make either a .par file or a ref_frames.dat file of the structure. Both of these files can be used as input for FRETmatrix. See the input folder for examples.


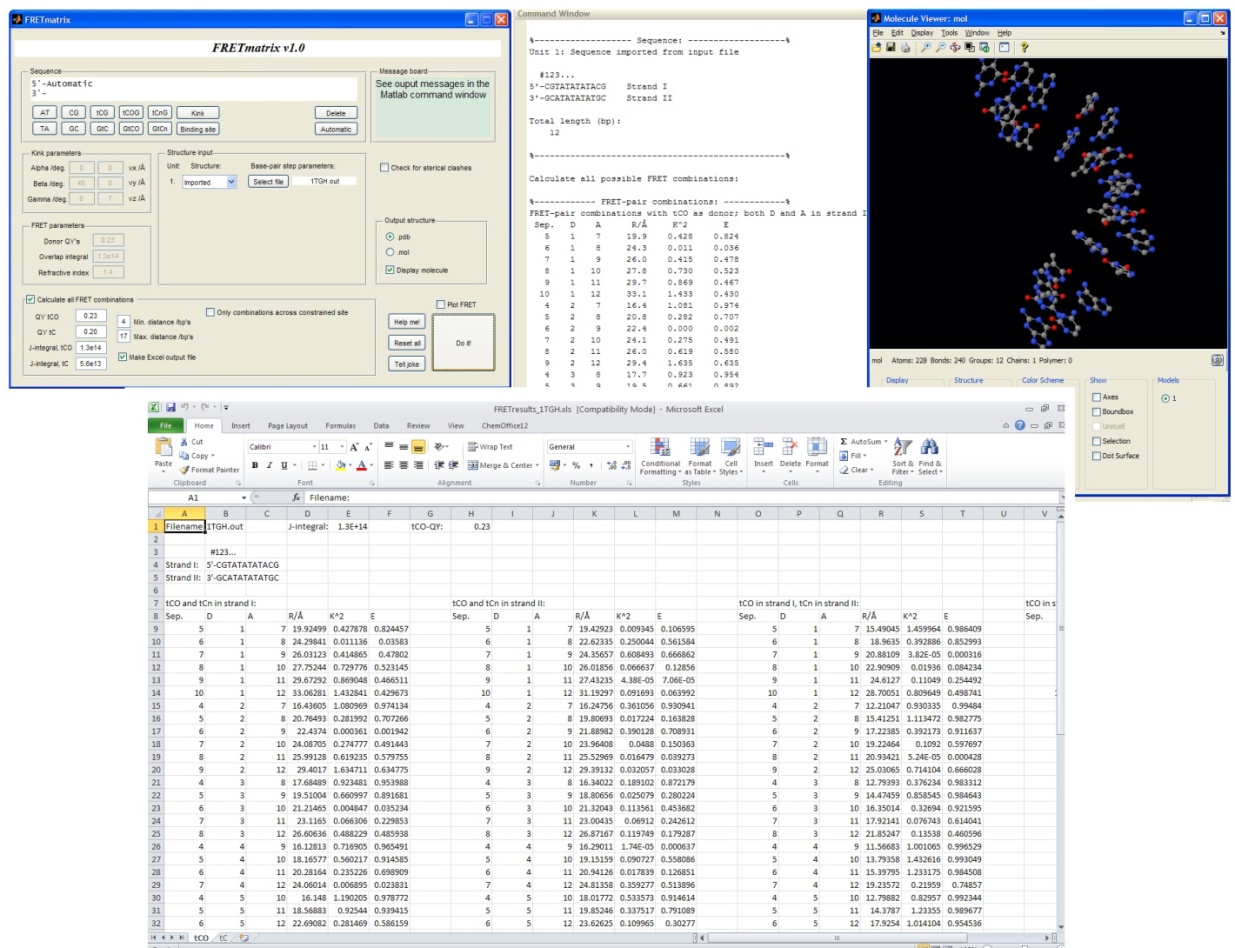


Figure 18. Screenshots from a working example 2. Lower insert shows output excel file.

Part II:

Analysis of base-base FRET

# Introductory comment

The FRETmatrix analysis program performs a global analysis of the time-resolved donor intensity decays from multiple FRET pairs in a base-base FRET experiment. To allow for flexibility the analysis part of FRETmatrix is not accompanied by a user interface but is run via the RunProgram.m file. Preparation of data for the analysis is automated by running the PrepareDecays.m script. If large chi-square surfaces will be run the decays can be pre-fit using MakeStoredFits.m prior analysis to save computational time.

# 1 Prepare decays for analysis

1) Name the decay files according to the positions of the donor (D) and acceptor (A) in the sequence. For example:

| D10A19.txt  D10.txt | Decay for donor at position 10 and acceptor at position 19.  Reference decay for donor at position 10. |
| --- | --- |

If in doubt of the numbering, run a single calculation of the sequence using the user interface in Part I above and deduce the D and A positions from the results displayed in the MATLAB command window.

2) The decay files must be set up in the following format:

| Some lines of text  t_decay I_decay t_IRF I_IRF  …  2.7052 14 2.7674 2194  2.7126 25 2.7747 3487  2.7199 27 2.7821 4801  2.7272 33 2.7894 6457  … |
| --- |

Here, t_decay contains the times associated with the decay intensity counts in I_decay, and t_IRF contains the times associated with the IRF intensity counts in I_IRF. Columns are separated by a tab. If the decay files are not automatically set up like this, they must be done so manually, e.g. using Excel. Examples are provided in the inputs/decays folder.

3) Open PrepareDecays.m. This script optimizes the decay parameters of each decay needed for the later analysis. The aim is to automatically determine values of time intervals and shifts of each decay. This is done by fitting the decays to an exponential decay model. The results are exported to a text file which is used by FRETmatrix in the FRET analysis part (below). The PrepareDecays.m will look something like this:

| % Specify files:  filenames = 'D7A14' 'D7A15' 'D7A20' 'D7A21' 'D9A15';  extension = '.dat';  folder = 'C:\FRETmatrix\inputs\decays\B-DNA';  Studyname = 'B-DNA';    % Settings:  I_tstart = 10; % Start the time when intensity reaches this count  I_tend = 20; % End the time when intensity reaches this count  skiplines = 13; % Skip these first lines in each datafile (being comments)  plotfit = 'N'; % Plot final fits? 'Y' or 'N'  Save_results = 'Y'; % Saved as .txt in same folder as the decays  L = 10; % Interpolation factor. If L=10, then shift=10 is 1 channel shift.  shiftrange = [0 150]; % The shift range investigated    % Start guesses:  alpha123 = [1; 0.05; 10]; % Pre-exponential factors  tau123 = [3.0; 5; 0.1]; % Lifetimes    % Set upper and lower boundaries [min max]  a1 = [0 100]; % Pre-exponential factor 1  a2 = [0 100];  a3 = [0 0];  t1 = [0 inf]; % Lifetime 1  t2 = [0 inf];  t3 = [1 1];    % Optimization options (can be increased for higher accuracy):  MaxFunEvals = 5000; % Maximum no. of function evaluations  MaxIter = 1000; % Maximum no. of iterations |
| --- |

Choose the filenames to be analysed and run the script (press F5 or type PrepareDecays from the MATLAB command window). The fitting will take some time depending on the number of files and the size of the shift range (Figure 19). A progressbar will show the estimated calculation time and results are continuously updated in the MATLAB command window.


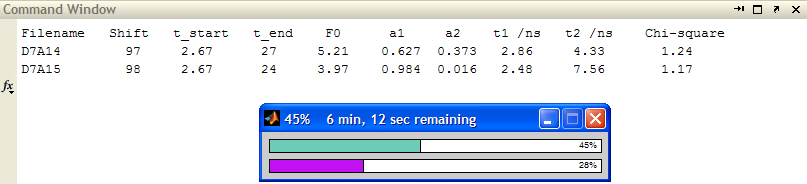


Figure 19. Running PrepareDecays.m

The output file, named ‘Studyname’_decaypar.txt, will look something like this:

| Filename Shift t_start t_end F0 a1 a2 t1/ns t2/ns Chi-square  D7A14 97 2.67 27 5.21 0.63 0.37 2.86 4.33 1.24  D7A15 98 2.67 24 3.97 0.98 0.02 2.48 7.56 1.17  D7A20 11 3.55 30 4.80 0.22 0.78 3.44 4.37 1.13  D7A21 32 3.56 29 4.64 0.68 0.32 3.78 4.61 1.10  D9A15 86 2.70 17 0.61 0.99 0.01 0.91 4.13 1.95 |
| --- |

Here, Shift is a channel shift between the decay and the IRF (actually channelshift*L), t_start and t_end is the start and end times, respectively, F0, a1, and a2 are pre-exponential factors, t1 and t2 are the fitted lifetimes, and the Chi-square value is for the fit obtained with the optimized parameter values. Note that in some cases the time-intervals chosen by the fitting routine is not optimal and must be manually set instead. *In general, the fits obtained using the optimized parameters should always be thoroughly checked before continuing with the analysis, as the decay parameters will affect the results of the analysis if not properly set.* Check the resulting fits by setting plotfit = 'Y'.

# 2 Speeding up analysis: Store pre-fit chi squares

This step is optional but can be used to save computational time during the chi-square FRET analysis if large chi-square surfaces will be calculated. The purpose here is to pre-fit all decays for different values of the κ^2^/*R*^6^ ratio prior the actual FRET analysis. The fitted chi-squares from this step are then called during the FRET analysis when a given κ^2^/*R*^6^ is encountered for each D-A pair. This eliminates the need to fit the decays over and over again when running the chi square surfaces during a FRET analysis, however, running all the pre-fits requires some computational time invested. If this step is not chosen, FRETmatrix will fit all the decays during a chi-square surface FRET analysis run.

1. Open MakeStoredFits.m. The input for the script will look something like this:

| % Specify study:  Studyname = 'B-DNA'; % Position decays: FRETmatrix/inputs/decays/Studyname  Samples = 'D7A14 D7A15 D7A20 D7A21 D9A15'; % D-A combinations for analysis.  file_extension = '.dat';    % Chi square parameters:  M = 2500; % No. of calculated chi-squares for each decay  E_lower = 0; % Lower efficiency cut off  E_upper = 0.8; % Upper efficiency cut off    % Donor reference lifetimes in same order as in sequence:  tau_d = [4.32 4.35 4.55]; % /ns    % Fraction of donor components in decays. Same donor order as in Sequence  frac_Ds = [ 1 1 1 1 1 1 1 1 1]; % Fraction of donor component in decay  tauIMP = 12.01; % Lifetime of possible impurity  frac_FRET = [0.996 0.996]; % Fraction of donors coupled to acceptor    % FRET parameters:  Jint = 1.3e14; % Overlap integral M.1cm.1nm4  ny = 1.4; % Refractive index  Qd = 0.23; % Donor quantum yield    % Optimization settings:  decayoptim_Tol = [0.01 0.001]; % [TolFun TolX] Fun: ChiSq, X: F0 & A  Decay_MaxFunEvals = 10000; % Maximum no. of function evaluations  Decay_MaxIter = 5000; % Maximum no. of iterations in decay fitting    % Export results to .txt file?  save_storedchis = 'Y'; % Will be saved in the decay folder |
| --- |

1. Specify samples and decay parameters and run the script by pressing F5. The script will call the shift and time-intervals from the decaypar.txt file produced as described above. Here, all decays will be fit at M different FRET efficiencies ranging from E_lower to E_upper. An output file called ‘Studyname’_storedchis.txt containing all the fit chi-square values and the parameters used to produce the fits will be saved to the decays folder. This output file is then called when running the FRETmatrix chi-square surface analysis (below). If the stored chis are used, the FRET parameters used to calculate the pre-fit chi-squares, specified in the storedchis.txt file, will automatically be imported in the FRET analysis. The resulting storedchis.txt file will look something like this:

| 22  Studyname: B-DNA  Samples:  D7A14 D7A15 D7A20 D7A21 D9A15  M = 2500  tau_d:  4.32 4.35 4.55  tauIMP:  12.01  frac_Ds:  1.000 1.000 1.000 1.000 1.000  frac_FRET:  0.996  Jint:  1.30e+014  ny:  1.40  Qd:  0.230 0.230  Stored chi-square values:  k^2/R^6 D7A14 D7A15 D7A20 D7A21 D9A15 0.000000e+000 49.874270 266.638946 2.008859 5.506675 561.976776  4.662394e-013 49.699960 266.301566 1.992618 5.450659 561.840096  9.327776e-013 49.508359 265.946423 1.966164 5.400735 561.700064  1.399615e-012 49.331958 265.602516 1.941512 5.352094 561.560488  1.866751e-012 49.157133 265.260176 1.922117 5.292344 561.421369 2.334187e-012 48.979170 264.913367 1.900920 5.242812 561.287494  2.801923e-012 48.796988 264.563507 1.876595 5.192448 561.152260  … |
| --- |

# 3 Run base-base FRET analysis

Open RunProgram.m. This file contains all variables and choices that must be specified when running the FRETmatrix analysis. This file is thus equivalent to the user interface in part I, and can also be used instead of the user interface in part I. The RunProgram.m will look something like this:

| %% Structure and FRET pairing input parameters    % Sequence in sequential base-pairs: (Write: kink, site or space for separating structural units). Tip: You can always write Automatic;  % Example:  % Sequence = [CG GC AT TA] corresponds to:  % 5'-CGAT Strand I  % 3'-GCTA Strand II    Sequence = [tCOG AT tCOG AT tCOG AT AT GtCn GtCn AT CG GC AT GtCn GtCn AT];    % Structure information:  Structure = 'B'; % 'A', 'B'-form or 'I' for imported  filename = 'None'; % For structure import: .dat, .par, .out    % Display structure in Matlab? 'Y' or 'N'.  display_mol = 'N'; % Displays optimized molecular structure after analysis    % Kink parameters. If no. of parameters specified < no. of kinks, then all  % kinks in the structure are defined by the same set of parameters:  % Kink # [1 2...; ]. Won’t be used for analysis part. See below.    KinkPar = [0;... % Alpha /deg  45;... % Beta /deg  0;... % Gamma /deg  0;... % vx /Å  0;... % vy /Å  6]; % vz /Å    % FRET parameters:  Qd = [0.23 0.23 0.23]; % Donor quantum yields  Jint = 1.3e14; % Overlap integral    % Automatically calculate all FRET combinations?  Calc_All = 'N'; % Y or N  SepCutOff = [4 15]; % FRET-pair separation range (in bp)  ConstrainSite = [10 11]; % Left and right bp next to the site    %% Additional input parameters:  moloutfile = 'pdb'; % Structure output file: 'pdb' or 'mol'  checkclashes = 'N'; % Check for sterical clashes? For analysis, see below.  vdwradius = 1.3; % Van der Waals radii for steric clash detection /Å  bspar = 'seq'; % Base-pair step parameters: seq, avg  ny = 1.4; % Refractive index    % For calculating all FRET combinations:  MakeExcel = 'Y'; % Make excel file with output data? 'Y' or 'N'  QY_tCO = 0.23; % Quantum yield of tCO  QY_tC = 0.20; % Quantum yield of tC  J_tCO = 1.3e14; % Overlap integral with tCO as donor  J_tC = 5.6e13; % Overlap integral with tC as donor    % Chi-Square analysis? 'Y' or 'N'.  Analyse = 'Y'; % Specify input parameters below |
| --- |
| %% Input parameters for fitting the decays:    % Specify study:  Studyname = 'B-DNA'; % Decay folder: FRETmatrix/inputs/decays/Studyname  Samples = 'D7A14 D7A15 D7A20 D7A21 D9A15'; % D-A combinations for analysis.  file_extension = '.dat'; % Suffix of decay files    % Donor reference lifetimes in same order as in sequence:  tau_d = [4.32 4.35 4.55];    % Fraction of donor components in decays. Same donor order as in Sequence  frac_FRET = [0.99 1]; % Fraction donors w. FRET  frac_Ds = [ 1 1 1 1 1 1 1 1 1 ]; % Fraction of donor component in decay  frac_don = [0.99 1]; % Fraction of donor component in decay  tauIMP = 12.01; % Lifetime of possible impurity  use_frac_Ds = 'N'; % Use specific values of frac_don? defined in frac_Ds  % Decay parameter file:  decayparfile = 'B-DNA_decaypar.txt'; % Positioned in inputs folder. |
| %% Input parameters for chi-square analysis:    % Run several chi-square surfaces and average?  chiruns = 1; % No. of chi-square surface runs  Save_ChiSqSumMatrix = 'N'; % Save the calculated chi-square surfaces?  PlotAvg_ChiSqSumMatrix = 'N'; % Plot the averaged chi-square surface?    % Sampling parameters and chi-square analysis  N = 1000; % No. of samples drawn from distribution  % Lower and upper bounds in optimization:  % Insert values as: [start end #steps]. If #steps = 1, the parameter will % be optimized within the specified start-end interval at each coordinate % on the calculated chi-square surface.  % Base probe parameters:  FWHMtheta_lu = [0 20 4]; % Theta-FWHM /deg.  FWHMphi_lu = [0 20 4]; % Phi-FWHM /deg.  bendA_lu = [25 25 1]; % Acceptor bending angle /deg.  bendD_lu = [8 8 1]; % Donor bending angle /deg.  J_lu = [1.3e14 1.3e14 1]; % Overlap integral /M-1cm-1nm4    % Kink parameters:  alpha_lu = [0 0 1]; % /deg.  beta_lu = [0 0 1]; % /deg.  gamma_lu = [0 0 1]; % /deg.  vx_lu = [0 0 1]; % /Å  vy_lu = [0 0 1]; % /Å  vz_lu = [0 0 1]; % /Å    % Type of distributions: 'LJ'/'H' is Len. Jones or Harmonic, respectively.  thetaType = 'LJ'; % Potential in theta direction (in plane).  phiType = 'H'; % Potential in phi direction (out of plane)    % Use pre-fitted chi-square values from make_storedchis.m  use_storedchis = 'Y'; % 'Y' or 'N'  storedchis_filename = 'B-DNA_storedchis_ex.txt'; % Located in decays folder    % Geometrical constraints in optimization? 'Y' or 'N'.  clash_constraint = 'N'; % Use sterical clash constraint in analysis  neighbdist_constraint = 'N'; % Use distance constraint in analysis  longstrand = 1; % The strand with extra bases (1 or 2)  vdwradius = 1.3; % van der Waals radii  neighbdistmax = 11; % Max. neighbour-distances in same strand /Å  ChiSqPenalty = 60; % Chi-square penalty for violated constraint  sa_constraints = 'Y'; % Use simulated annealing for first optimization step  % Plots? 'Y' or 'N'  plot_chisqsurf = 'Y'; % Plot all calculated chi-square surfaces?  plot_decay = 'N'; % Plot fitted decays?  plot_FRET = 'N'; % Plot resulting FRET efficiencies?  plot_sampling = 'N'; % Plot sampled distribution?  savefits = 'N'; % Save fitted decays?    % Matlab Pooling? 'Y' or 'N'  pooling = 'N'; % If possible, activate for faster distribution optimization    % Optimization settings:  Joptim_Tol = [0.05 0.01e14]; % [TolFun TolX] Fun: ChiSqSum, X: Jint  ChiOpt_TolX = 0.001; % TolX: FWHM, bend  ChiOpt_TolFun = 0.001; % TolFun: ChiSqSum  KinkOpt_Tol = [0.01 0.01]; % [TolFun TolX] Fun: ChiSqSum, X: KinkPar  decayoptim_Tol = [0.01 0.001]; % [TolFun TolX] Fun: ChiSq, X: F0 & a,b  Decay_MaxFunEvals = 5000; % Max function evaluation in decay fitting  Decay_MaxIter = 1000; % Max iterations in decay fitting    % Simulated annealing algorithm settings (see also Matlab saoptimset help):  sim_anneal_optim = 'N'; % Optimize kink using simulated annealing?  saLimit = 30; % Stop optimization when the chisq reaches this value  saTolFun = 0.001; % Stop optimization when change in chisq < saTolFun  saTimeLimit = 3*60; % Stop optimization after saTimeLimit seconds  saStallTimeLimit = 500; % Stop iteration after saStallTimeLimit seconds  saDisplay = 'iter'; % Display data from optimization in Matlab workspace.  saPlot = 'N'; % Plot data computed by the simulated annealing algorithm?  saTempFcn = @temperatureexp; % Decrease T according to saTempFcn function  saInitialTemp = 200; % Initial temperature  % Print results to file? 'Y' or 'N'.  Resultsfile = 'N'; % Will be written to Studyname_analysis.txt |

Here, the red section is analogues to the user interface above, while the blue sections contain the parameters used to run the chi-square analysis. The input parameters are described in the comments shown above. Some additional notes of clarification:

| **Running analysis** | To run a chi-square analysis Analyse must be set to 'Y', else the program will only perform a single point calculation using the parameters specified in the red section. |
| --- | --- |
| **Study name** | The study name is used as an identifier by FRETmatrix to keep track of more than one studies. It has an impact in a number of different ways:  1) When running an analysis, FRETmatrix will look in the folder: FRETmatrix/inputs/decays/’Studyname’ for the decays.  2) The decaypar.txt file will/must be named ‘Studyname’-_decaypar.txt. and be placed in the decays/’Studyname’ folder.  3) The pre-fit chi-squares file will be called ‘Studyname’-_storedchis.txt and must be placed in the decays/’Studyname’ folder. |
| **Specifying decays** | Using the filename convention described above, the program automatically match a decay file specified in the Samples input with a donor-acceptor position in the Sequence. The names do not need to be specified in any specific order. |
| **Choosing D-A combinations** | Any sub-group of donor-acceptor pairs out of the total number of possible combinations can be specified for analysis in the Samples input. In case not all FRET combinations were measured, or not all decays are considered to be well resolved for the analysis, the program will only use the combinations specified in Samples and automatically pair the chosen D-A combinations with the corresponding parameters specified in the decaypar.txt file. |
| **Presence of impurity or non-coupled donors** | The presence of even very small amounts of uncoupled donors or an emitting impurity in the sample will have an impact on the fitted decays when the FRET efficiency is high (short donor lifetimes). The fraction of donors in the sample (1-‘fraction of impurity’) is set by the frac_Ds (for choosing specific values) or the frac_don (for optimizing the value). The lower and upper boundary of fraction of donors coupled to an acceptor is set by the frac_FRET. |
| **Running several chi-square surfaces** | The program will run the chi-square surface using the specified parameters “chiruns” times. If Save_ChiSqSumMatrix = ‘Y’ all the calculated surfaces will be saved in the outputs folder. If PlotAvg_ChiSqSumMatrix = ‘Y’ the calculated surfaces will be saved to the outputs folder and the surface averaged from all runs will be made, saved and plotted. |
| **Setting parameters for chi-square analysis** | The parameter intervals are set under “Lower and upper bounds in optimization” in the blue section above. Parameters are set according to *_lu = [lower upper #steps]. E.g. the setting alpha_lu = [0 30 4] will make 4 alpha steps from 0° to 30° (i.e. 0°, 10°, 20°, and 30°), whereas alpha_lu = [0 30 1] will optimize the value of α within the lower and upper boundaries (here 0°-30°). The program automatically identifies what parameters are chosen as the *x* and *y* of the χ^2^ surface being two parameters where #steps > 1. At each coordinate on the surface, the parameters not included in the chi-square surface are optimized within the chosen lower and upper conditions. Also one dimensional chi-square surfaces can be run if only one parameter is set at #steps > 1. If #steps = 1 for all parameters the program will begin a global optimization of all parameters. |
| **Type of distribution** | Defines the appearance of the probability distributions representing in-plane and out-of-plane base motion. In the program a harmonic potential is simulated by sampling a Gaussian distribution. |
|  |  |
| **Imposing geometrical constraints in analysis** | When having geometrical constraints activated in the analysis all parameter values resulting in a nucleic acid structure in violence with the chosen constraints are automatically discarded in the analysis and the χ^2^ is set to a large value (the ChiSqPenalty). In the final chi-square plot, those parameter values that resulted in a violence of one of the imposed constraints will automatically be associated with the highest chi-square value on the surface.  The clash_constraint discards structures in which there is a sterical clash between two atoms in different bases. A sterical clash is when the interatomic distance is *r* < 2**vdw*, where *vdw* is the atomic van der Waals radius. The neighbdist_constraint imposes a maximum distance between two neighbouring bases in a strand, set by the neighbdistmax value.  In some cases the gradient based algorithm may get stuck at the ChiSqPenalty if the parameter value start guesses themselves are in violence with the constraints. To avoid this, an initial simulated annealing algorithm[^14^](#_ENREF_14) optimization step can be chosen by setting sa_constraints = ‘Y’. This first step will find a set of suitable starting conditions for the gradient based parameter optimization algorithm. |
| **Plotting** | plot_chisqsurf: Will plot the calculated chi-square surface. If chiruns > 1 each calculated surface will be plotted if this setting is activated. If only the averaged surface is wanted, set PlotAvg_ChiSqSumMatrix = ‘Y’ and plot_chisqsurf = ‘N’.  plot_decay: Will plot all the fitted decays. This setting can only be chosen using one single specific set of parameters (i.e. not when an analysis is run).  plot_FRET: Will plot the FRET efficiency graph. Can only be chosen when using one single specific set of parameters.  plot_sampling: Will plot the sampled dipole distributions of the first donor and last acceptor in the sequence in Cartesian space. The coordinates of directions can be directly compared within the coordinate frame of the global structure. To see the coordinate axes of the structure, set ‘Axes’ on in the molviewer when visualizing the molecule.  savefits: Will save the measured and fitted decays in two matrices in the outputs folder for later plotting: allmeasdecays.mat and decayfitresults.mat. The saved decays and fits can then be plotted one by one using the plotsaveddecays.m script located in the ‘Calls’ folder. This setting should only be chosen when using one single specific set of parameters. |
| **MATLAB pool** | See the MATLAB instructions for a description. This setting activates parallel computing on a pool of workers (e.g. more than one processor). In some cases it can speed up the optimization of the parameters: θ-FWHM, φ-FWHM and the bending angles. |
| **Balancing speed and accuracy of fit** | The “Optimization settings” provide a means to balance the speed and accuracy of the optimization. In general, the TolFun and TolX values provide more accurate fits at lower values while the MaxFunEvals and MaxIter provide more accurate fits at larger values. |
| **Choosing optimization algorithm** | When optimizing all kink parameters at once choosing the simulated annealing optimization algorithm[^14^](#_ENREF_14) may be advantageous if the chi-square surface is complex. This algorithm is chosen by setting sim_anneal_optim = ‘Y’. However, whereas this algorithm may find the global minimum it does so very slowly. If in doubt, disable simulated annealing. See the Matlab manual for a description of simulated annealing optimization settings. |
| **Resultsfile** | This will export all details of the fits from each step in the chi-square surface analysis to a .txt file located in the ‘outputs’ folder named ‘Studyname’_analysis.txt. |

### Working example 1: Run chi-square surface with θ-FWHM and φ-FWHM as parameters.

1. Open RunProgram_Example1.m.
2. Run a chi-square surface using the predefined settings by pressing F5.

This will run a chi-square surface using the θ-FWHM and φ-FWHM as the two surface parameters. The samples are a set of donor-acceptor combinations in B-DNA located in the inputs/decays/B-DNA folder. The input sequence is:

%---------------------------%

(X,Y,Z = tC,tCO,tCn)

#123...

5'-CGATCAYAYAYAAGGACGAGGATAAGGAGGAGG Strand I

3'-GCTAGTGTGTGTTZZTGCTZZTATTCCTCCTCC Strand II

Total length (bp):

33

%---------------------------%

In this example, four steps of each surface parameter are calculated in the interval from 0° to 20° (i.e. at values 0°, 6.67°, 13.3°, and 20°) yielding sixteen coordinates on the resulting chi-square surface (Figure 20). Points in between the sixteen steps are interpolated. In this example, the other parameter values are not optimized at each coordinate on the surface since the upper and lower boundaries are set to be equal. Change the parameter intervals by setting the *_LU parameters. This example uses pre-fit decays and thus requires the B-DNA_storedchis_ex.txt (made by MakeStoredFits.m) to be located in the decays folder. The resulting chi-square surface is an average of 3 runs.


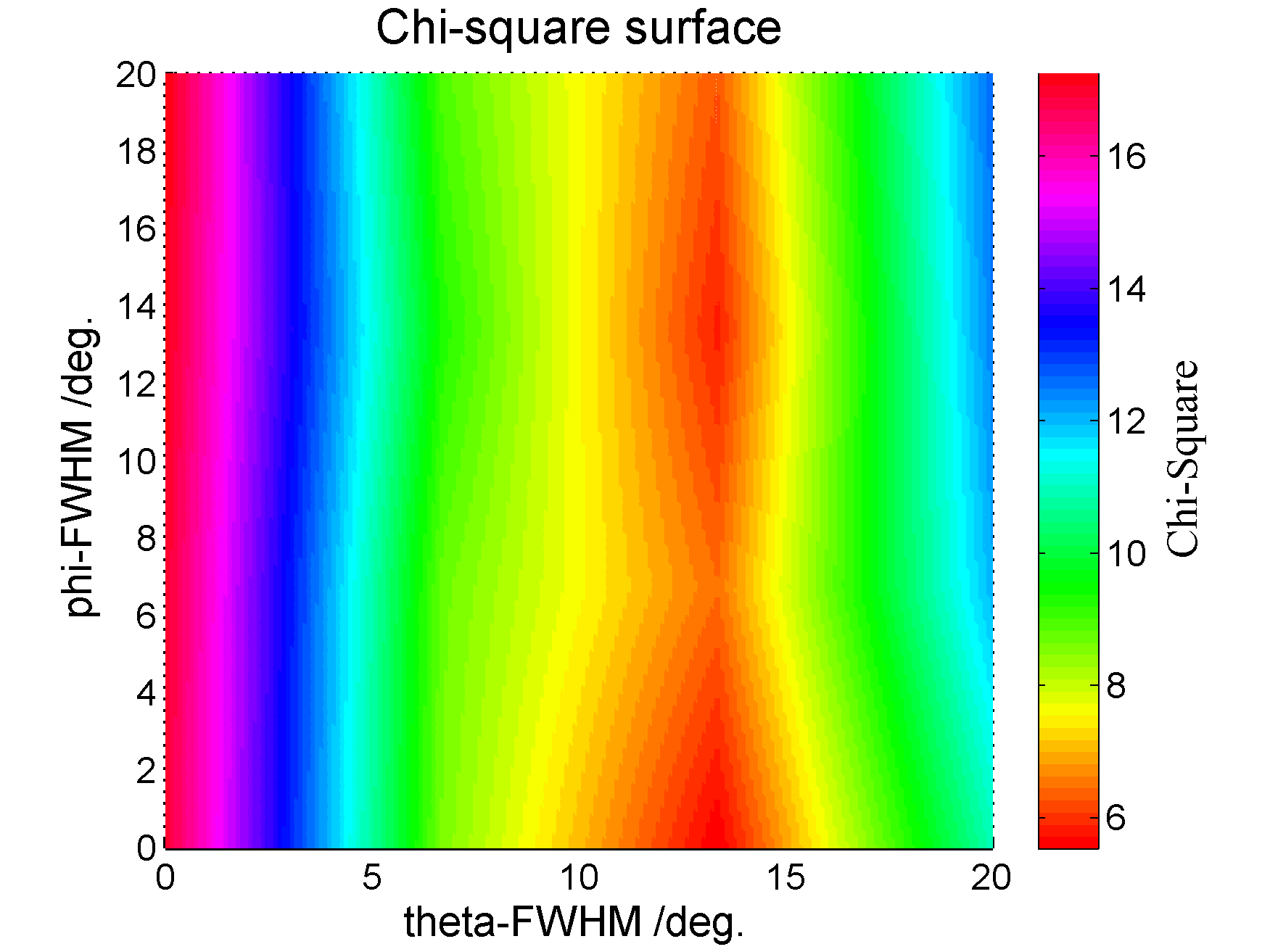


Figure 20. Result from a run of Working example 1.

### Working example 2: Optimize kink parameters without constructing chi-square surface

Although it is possible to run a simple optimization, this example is not advised for obtaining the parameter values since there is a high risk that the optimization algorithm gets stuck in a local minimum. In addition, the simulation is based on random sampling and the found parameters will therefore differ from one optimization to the next. In some cases it may be a useful first hint of where to look on the chi-square surface (Working example 1 and 3).

1. Open RunProgram_Example2.m.
2. Run a parameter optimization using the predefined settings by pressing F5.

In this example the same samples as in Working example 1 are analysed. However, a kink has now been defined in the sequence in between bp 12 and bp 13. The sequence is now:

#123...

5'-CGATCAYAYAYA AGGACGAGGATAAGGAGGAGG Strand I

3'-GCTAGTGTGTGT TZZTGCTZZTATTCCTCCTCC Strand II

The structure is thus modelled by the program as two structural B-DNA units separated by a kink with the kink being a regular base-pair. The intrinsic probe parameters are set to: θ-FWHM = 13°, φ-FWHM = 2°, tC_nitro_-φ_b_ = 25°, tC^O^-φ_b_ = 8°. The sterical clash constraint has been enabled in the optimization.

A resulting output in the MATLAB command window will look like this:

…

No parameters have been specified for a chi-square analysis

### %-----------------------%

### Global Chi-square: 3.415

### Base probe parameters:

### thetaFWHM = 13.00;

### phiFWHM = 2.00;

### bendA = 25.00;

### bendD = 8.00;

### J = 130000000000000.00;

### Kink parameters:

### alpha = 40.80;

### beta = 1.81;

### gamma = 0.00;

### vx = 1.41;

### vy = -2.31;

### vz = 2.86;

### %-----------------------%

Note: The simulated annealing algorithm[^14^](#_ENREF_14) can be chosen for this example if the chi-square surface is complex. Set sim_anneal_optim = 'Y' to activate simulated annealing.

### Working example 3: Run chi-square surface of kink parameters.

1. Open RunProgram_Example3.m.
2. Run a chi-square surface using the predefined settings by pressing F5.

This will run a chi-square surface of the same setup as in Working example 2. The *x*- and *y*-values of the surface are the DNA-bending angle, β, and the helical rise, *v*_z_, respectively, each calculated at four steps (Figure 21). The other parameters are optimized within the specified intervals which is why this example requires considerably longer computational time than Working example 1. The sterical clash constraint is enabled which is why the chi-square value is observed to drastically increase at *v*_z_ values below 3 Å. Setting display_mol = ‘Y’ will show the structure corresponding to the minimum found on the calculated chi-square surface which in this case is a regular B-DNA helix (Figure 22).

| 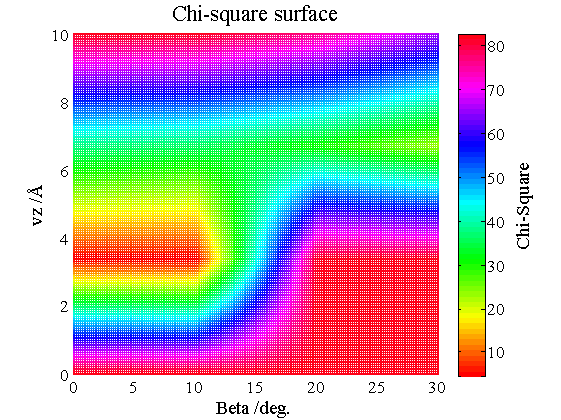  **Figure 21. Resulting chi-square surface of one run of Working Example 3.** | 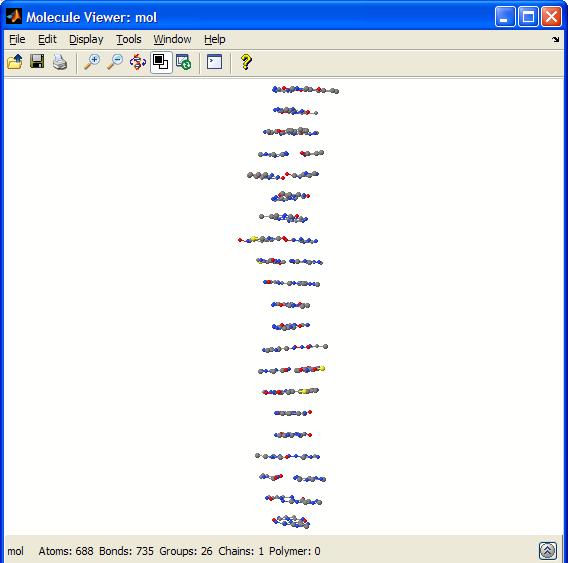  Figure 22. Resulting output structure from Working example 3. |
| --- | --- |

# Implementing a new base probe in the program

This is done using the MakeBP.m script located in the calls folder. A punctuated protocol is supplied in the help intro of the script.

# User Guide References

(1) Arnott, S.; Hukins, D. W. L. *Biochemical and Biophysical Research Communications* **1972**, *47*, 1504.

(2) Arnott, S.; Hukins, D. W. L. *J. Mol. Biol.* **1973**, *81*, 93.

(3 )Olson, W. K.; Bansal, M.; Burley, S. K.; Dickerson, R. E.; Gerstein, M.; Harvey, S. C.; Heinemann, U.; Lu, X. J.; Neidle, S.; Shakked, Z.; Sklenar, H.; Suzuki, M.; Tung, C. S.; Westhof, E.; Wolberger, C.; Berman, H. M. *J. Mol. Biol.* **2001**, *313*, 229.

(4) Lu, X. J.; Olson, W. K. *Nucleic Acids Research* **2003**, *31*, 5108.

(5) Zheng, G. H.; Lu, X. J.; Olson, W. K. *Nucleic Acids Research* **2009**, *37*, W240.

(6) Preus, S.; Börjesson, K.; Kilså, K.; Albinsson, B.; Wilhelmsson, L. M. *J. Phys. Chem. B.* **2010**, *114*, 1050.

(7) Sandin, P.; Börjesson, K.; Li, H.; Mårtensson, J.; Brown, T.; Wilhelmsson, L. M.; Albinsson, B. *Nucleic Acids Research* **2008**, *36*, 157.

(8) Sandin, P.; Wilhelmsson, L. M.; Lincoln, P.; Powers, V. E. C.; Brown, T.; Albinsson, B. *Nucleic Acids Research* **2005**, *33*, 5019.

(9) Kirkpatrick, S.; Gelatt, C. D.; Vecchi, M. P. *Science* **1983**, *220*, 671.

End of user guide

# SUPPLEMENTARY DATA

Supplementary Data 1. Base-pair step parameter matrix of pdb entry 1TGH, the complex formed between the TATA binding protein and its DNA target ([13](#_ENREF_13)). The data was obtained using w3DNA ([11](#_ENREF_11)).

Local base-pair step parameters

step Shift Slide Rise Tilt Roll Twist

1 CG/CG 0.21 0.65 2.95 0.30 8.52 35.43

2 GT/AC 0.16 -0.53 3.58 -3.24 -1.37 41.06

3 TA/TA 0.46 -0.23 5.08 5.75 45.10 18.59

4 AT/AT -1.87 -0.24 3.69 1.58 24.83 16.06

5 TA/TA 0.45 2.37 3.17 0.06 7.94 25.61

6 AT/AT 0.80 0.95 3.14 -0.16 21.26 1.46

7 TA/TA 0.02 2.26 3.97 0.51 31.05 27.83

8 AT/AT 0.58 0.04 3.23 -1.79 13.98 20.50

9 TA/TA -0.08 -0.81 5.49 0.89 50.70 16.68

10 AC/GT -0.74 0.09 2.78 4.55 13.80 15.74

11 CG/CG -0.59 1.36 3.27 -2.18 3.07 32.12

**Supplementary Data 2.** Simulated FRET characteristics of model systems (Figure 3 in paper). Sep. denotes donor-acceptor separation in base-pairs. D and A is donor and acceptor positions, respectively, in the sequence. R is the dipole center-center distance, K^2 is *κ*^2^ and E is the FRET efficiency.

B-DNA. Donor positioned in strand I, acceptor in strand II.

%------------ FRET-pair combinations: ------------%

Sep. D A R/Å K^2 E

0 1 2 5.2 0.311 1.000

1 1 3 6.7 0.003 0.952

2 1 4 9.8 0.483 0.997

3 1 5 13.5 0.760 0.988

4 1 6 17.4 0.487 0.924

5 1 7 21.1 0.086 0.402

6 1 8 24.6 0.049 0.133

7 1 9 27.9 0.484 0.415

8 1 10 31.0 0.921 0.417

9 1 11 34.0 0.784 0.258

10 1 12 37.1 0.219 0.055

11 1 13 40.3 0.021 0.003

12 1 14 43.6 0.489 0.047

13 1 15 47.1 0.950 0.057

14 1 16 50.6 0.754 0.030

15 1 17 54.2 0.187 0.005

%-------------------------------------------------%

B-DNA. Donor positioned in strand II, acceptor in strand I:

%------------ FRET-pair combinations: ------------%

Sep. D A R/Å K^2 E

0 1 2 8.3 0.156 0.997

1 1 3 10.7 0.247 0.991

2 1 4 13.0 0.086 0.923

3 1 5 15.4 0.047 0.706

4 1 6 18.0 0.554 0.919

5 1 7 20.8 0.898 0.884

6 1 8 24.0 0.489 0.639

7 1 9 27.4 0.020 0.031

8 1 10 31.0 0.213 0.141

9 1 11 34.6 0.755 0.231

10 1 12 38.2 0.893 0.165

11 1 13 41.6 0.452 0.056

12 1 14 44.9 0.021 0.002

13 1 15 48.1 0.199 0.011

14 1 16 51.2 0.777 0.029

15 1 17 54.4 0.964 0.025

%-------------------------------------------------%

A-RNA. Donor in strand I, acceptor in strand II:

%------------ FRET-pair combinations: ------------%

Sep. D A R/Å K^2 E

0 1 2 6.8 0.395 1.000

1 1 3 7.8 0.647 0.999

2 1 4 9.0 0.267 0.997

3 1 5 10.3 0.011 0.861

4 1 6 11.7 0.414 0.991

5 1 7 13.5 0.547 0.984

6 1 8 15.6 0.128 0.860

7 1 9 18.0 0.057 0.536

8 1 10 20.7 0.556 0.831

9 1 11 23.4 0.953 0.798

10 1 12 26.2 0.781 0.621

11 1 13 29.0 0.272 0.240

12 1 14 31.6 0.000 0.000

13 1 15 34.1 0.277 0.108

14 1 16 36.4 0.824 0.195

15 1 17 38.8 1.036 0.173

%-------------------------------------------------%

A-RNA. Donor in strand II, acceptor in strand I:

%------------ FRET-pair combinations: ------------%

Sep. D A R/Å K^2 E

0 1 2 6.4 0.170 0.999

1 1 3 7.8 0.223 0.999

2 1 4 10.0 0.042 0.966

3 1 5 12.7 0.036 0.853

4 1 6 15.6 0.256 0.925

5 1 7 18.5 0.431 0.881

6 1 8 21.3 0.371 0.734

7 1 9 23.9 0.139 0.339

8 1 10 26.3 0.000 0.000

9 1 11 28.7 0.175 0.178

10 1 12 30.9 0.540 0.298

11 1 13 33.2 0.687 0.261

12 1 14 35.6 0.414 0.123

13 1 15 38.0 0.053 0.012

14 1 16 40.6 0.075 0.011

15 1 17 43.3 0.490 0.049

%-------------------------------------------------%

DNA-TBP: No TBP

%------------ FRET-pair combinations: ------------%

Sep. D A R/Å K^2 E

4 1 6 18.7 0.279 0.819

6 1 8 24.6 0.050 0.135

8 1 10 30.9 0.923 0.419

10 1 12 37.2 0.003 0.001

3 2 6 15.4 0.047 0.708

5 2 8 21.6 0.801 0.843

7 2 10 27.4 0.400 0.395

9 2 12 34.6 0.752 0.231

%-------------------------------------------------%

DNA-TBP: With TBP bound

%------------ FRET-pair combinations: ------------%

Sep. D A R/Å K^2 E

4 1 6 18.3 0.658 0.923

6 1 8 19.0 0.393 0.853

8 1 10 22.9 0.019 0.084

10 1 12 33.1 1.433 0.430

3 2 6 17.9 0.032 0.398

5 2 8 19.8 0.017 0.164

7 2 10 24.0 0.049 0.150

9 2 12 34.2 0.042 0.017

%-------------------------------------------------%

# REFERENCES

1. Wilhelmsson, L.M., Sandin, P., Holmen, A., Albinsson, B., Lincoln, P. and Norden, B. (2003) Photophysical characterization of fluorescent DNA base analogue, tC. *J Phys Chem B*, **107**, 9094-9101.

2. Sandin, P., Borjesson, K., Li, H., Martensson, J., Brown, T., Wilhelmsson, L.M. and Albinsson, B. (2008) Characterization and use of an unprecedentedly bright and structurally non-perturbing fluorescent DNA base analogue. *Nucleic Acids Res*, **36**, 157-167.

3. Preus, S., Borjesson, K., Kilsa, K., Albinsson, B. and Wilhelmsson, L.M. (2010) Characterization of nucleobase analogue FRET acceptor tCnitro. *J Phys Chem B*, **114**, 1050-1056.

4. Sindbert, S., Kalinin, S., Nguyen, H., Kienzler, A., Clima, L., Bannwarth, W., Appel, B., Muller, S. and Seidel, C.A. (2011) Accurate distance determination of nucleic acids via Forster resonance energy transfer: implications of dye linker length and rigidity. *J Am Chem Soc*, **133**, 2463-2480.

5. Wozniak, A.K., Schroder, G.F., Grubmuller, H., Seidel, C.A. and Oesterhelt, F. (2008) Single-molecule FRET measures bends and kinks in DNA. *Proc Natl Acad Sci U S A*, **105**, 18337-18342.

6. Preus, S., Kilsa, K., Wilhelmsson, L.M. and Albinsson, B. (2010) Photophysical and structural properties of the fluorescent nucleobase analogues of the tricyclic cytosine (tC) family. *Phys Chem Chem Phys*, **12**, 8881-8892.

7. Lu, X.J., El Hassan, M.A. and Hunter, C.A. (1997) Structure and conformation of helical nucleic acids: rebuilding program (SCHNArP). *J Mol Biol*, **273**, 681-691.

8. El Hassan, M.A. and Calladine, C.R. (1995) The assessment of the geometry of dinucleotide steps in double-helical DNA; a new local calculation scheme. *J Mol Biol*, **251**, 648-664.

9. Arnott, S. and Hukins, D.W.L. (1972) Optimized parameters for A-DNA and B-DNA. *Biochem Bioph Res Co*, **47**, 1504-&.

10. Arnott, S. and Hukins, D.W. (1973) Refinement of the structure of B-DNA and implications for the analysis of x-ray diffraction data from fibers of biopolymers. *J Mol Biol*, **81**, 93-105.

11. Zheng, G., Lu, X.J. and Olson, W.K. (2009) Web 3DNA--a web server for the analysis, reconstruction, and visualization of three-dimensional nucleic-acid structures. *Nucleic Acids Res*, **37**, W240-246.

12. Olson, W.K., Bansal, M., Burley, S.K., Dickerson, R.E., Gerstein, M., Harvey, S.C., Heinemann, U., Lu, X.J., Neidle, S., Shakked, Z. *et al.* (2001) A standard reference frame for the description of nucleic acid base-pair geometry. *J Mol Biol*, **313**, 229-237.

13. Juo, Z.S., Chiu, T.K., Leiberman, P.M., Baikalov, I., Berk, A.J. and Dickerson, R.E. (1996) How proteins recognize the TATA box. *J Mol Biol*, **261**, 239-254.
